# Craniodental Morphology and Systematics of a New Family of Hystricognathous Rodents (Gaudeamuridae) from the Late Eocene and Early Oligocene of Egypt

**DOI:** 10.1371/journal.pone.0016525

**Published:** 2011-02-22

**Authors:** Hesham M. Sallam, Erik R. Seiffert, Elwyn L. Simons

**Affiliations:** 1 Department of Geology, Faculty of Sciences, Mansoura University, Mansoura, Egypt; 2 Department of Anatomical Sciences, Stony Brook University, Stony Brook, New York, United States of America; 3 Division of Fossil Primates, Duke Lemur Center, Durham, North Carolina, United States of America; University College London, United Kingdom

## Abstract

**Background:**

*Gaudeamus* is an enigmatic hystricognathous rodent that was, until recently, known solely from fragmentary material from early Oligocene sites in Egypt, Oman, and Libya. *Gaudeamus*' molars are similar to those of the extant cane rat *Thryonomys*, and multiple authorities have aligned *Gaudeamus* with *Thryonomys* to the exclusion of other living and extinct African hystricognaths; recent phylogenetic analyses have, however, also suggested affinities with South American caviomorphs or Old World porcupines (Hystricidae).

**Methodology/Principal Findings:**

Here we describe the oldest known remains of *Gaudeamus*, including largely complete but crushed crania and complete upper and lower dentitions. Unlike younger *Gaudeamus* species, the primitive species described here have relatively complex occlusal patterns, and retain a number of plesiomorphic features. Unconstrained parsimony analysis nests *Gaudeamus* and *Hystrix* within the South American caviomorph radiation, implying what we consider to be an implausible back-dispersal across the Atlantic Ocean to account for *Gaudeamus*' presence in the late Eocene of Africa. An analysis that was constrained to recover the biogeographically more plausible hypothesis of caviomorph monophyly does not place *Gaudeamus* as a stem caviomorph, but rather as a sister taxon of hystricids.

**Conclusions/Significance:**

We place *Gaudeamus* species in a new family, Gaudeamuridae, and consider it likely that the group originated, diversified, and then went extinct over a geologically brief period of time during the latest Eocene and early Oligocene in Afro-Arabia. Gaudeamurids are the only known crown hystricognaths from Afro-Arabia that are likely to be aligned with non-phiomorph members of that clade, and as such provide additional support for an Afro-Arabian origin of advanced stem and basal crown members of Hystricognathi.

## Introduction

**Figure 1:**
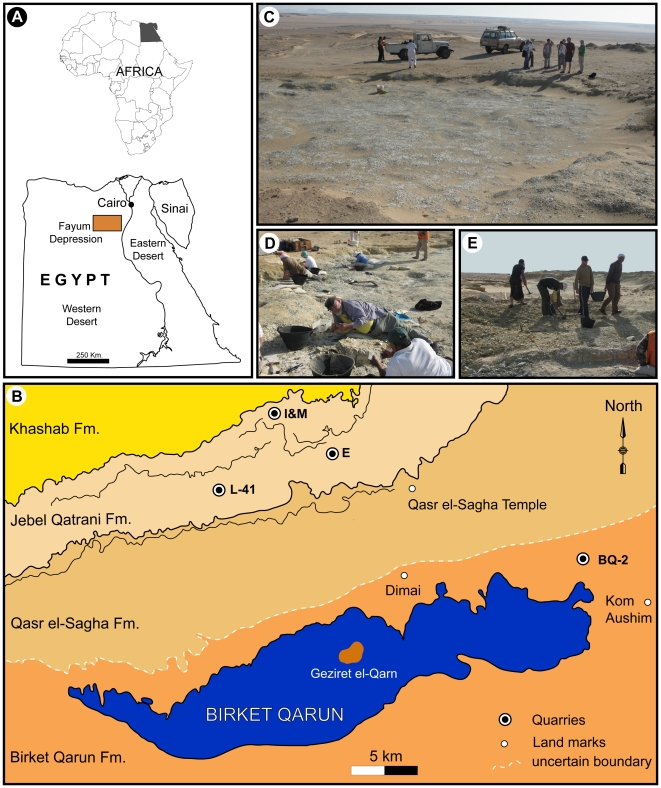
Location and overview of Quarry L-41. A, Location map of the Fayum Depression; B, close up map of Jebel Qatrani area, showing major land marks mentioned here; C, general view of Quarry L-41; D, quarrying process at L-41; E, workers exposing a new area of L-41.

**Figure 2:**
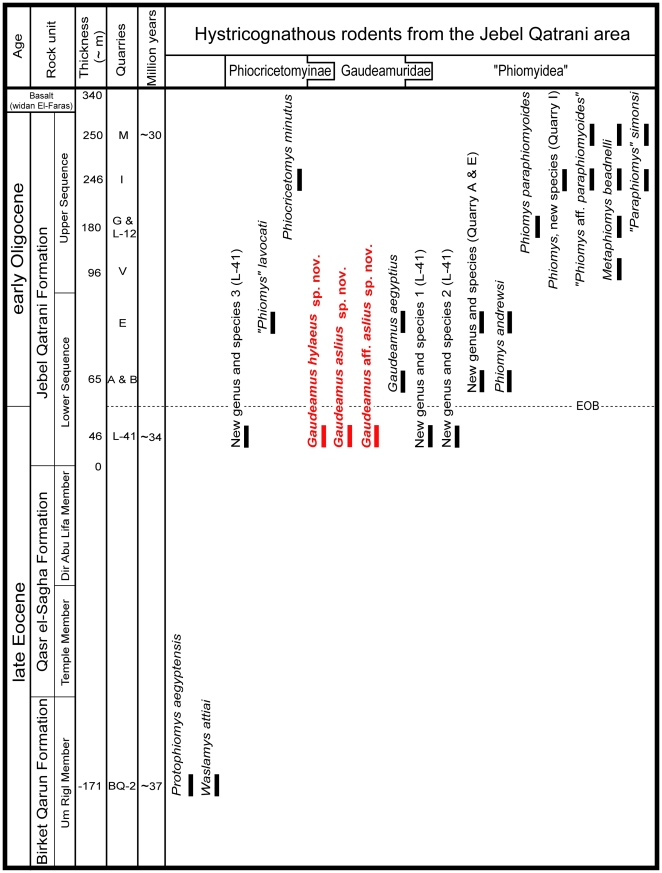
Stratigraphic positions and age estimates for major mammal-bearing fossil quarries, approximate position of Eocene-Oligocene boundary, following Seiffert [**6**], and distribution of hystricognathous rodents in the Jebel Qatrani area.

The Jebel Qatrani area of northern Egypt, which is located north-northwest of Birket Qarun (the largest lake in North Africa), preserves the richest terrestrial mammal-bearing Paleogene exposures in Egypt, if not the entire Afro-Arabian landmass [1], [2]. The eastern part of this protected area is situated approximately 85 km southwest of Cairo (Figure 1), and takes its name (Egyptian Arabic *gebel* = mountain, *qatrani* = tar) from the Widan el-Faras basalt, which unconformably caps the Oligocene succession. The geology of the Jebel Qatrani area is uncomplicated, consisting of a series of escarpments of late Eocene, early Oligocene, and Miocene age. Four formations, ranging in age from the late Eocene to the early Miocene, are exposed, and the most fossiliferous, the Jebel Qatrani Formation [3], previously known as the “Fluvio-Marine Series” [4] underlies the Miocene Khashab Formation and is late Eocene and early Oligocene in age [5], [6], [7]. The formation has been divided into two zones, previously referred to as the “Lower Fossil Wood Zone” and the “Upper Fossil Wood Zone” [8], now called the “lower sequence” and “upper sequence”, respectively [9]. The sediments of the Jebel Qatrani Formation consist primarily of variegated alluvial deposits [9], and overlie the nearshore marine and fluvial beds of the late Eocene Qasr el-Sagha Formation. Almost all of the area's major vertebrate fossil quarries, such as quarries A, B, E, V, I, M, and L-41 occur in the Jebel Qatrani Formation (Figure 2). Of these, only L-41 is likely to be late Eocene in age, and is probably very close to the Eocene-Oligocene boundary [6], [7].

Fieldwork in the Jebel Qatrani area in 1983 led to the discovery of the oldest and most productive quarry, Locality 41 (L-41) (Figure 1C–E). Annual excavations at L-41 undertaken by Duke University and the Cairo Geological Museum over the course of the subsequent 26 years have significantly increased the number of fossil vertebrate species known from the Fayum area. Species known from the L-41 are often represented not only by dental and mandibular remains, but also by complete crania and postcranial remains, though the latter are often badly crushed and distorted [10], [11], [12], [13].

Although hystricognathous rodents are often among the most abundant components of the Jebel Qatrani Formation's terrestrial mammal faunas, little has been published about the group. Despite being over four decades old, Albert Wood's analysis of Fayum rodents [14] remains the most significant publication on the group, though newer discoveries were subsequently dealt with in an unpublished doctoral dissertation [15]. The oldest hystricognaths from Egypt, ∼37 Ma *Protophiomys aegyptensis* and *Waslamys attiai*, were recently described by Sallam et al. [16], but more derived members of this rodent suborder have been discovered at numerous levels between the oldest (BQ-2, ∼37 Ma) and youngest (I and M, ∼30 Ma) major terrestrial mammal-bearing localities in the area. The rodents from the ∼34 Ma Quarry L-41 include evolutionarily intermediate forms that provide an important source of information not only for understanding the group's systematic position, but also for the evolution and biogeographic history of Hystricognathi.

Among other taxa, the extensive collection of hystricognathous rodents from L-41 includes numerous specimens of the genus *Gaudeamus*, including not only mandibles and maxillae, but also nearly complete crania. *Gaudeamus* is the most hypsodont rodent of the lower sequence, and bears molars with tall lophs and a highly derived, but relatively simple, occlusal pattern. *Gaudeamus* is intermediate in size when compared with other Fayum rodents such as the tiny genus *Phiocricetomys* and the relatively large genus *Metaphiomys* [17]. Remains of specimens that would ultimately be placed in the genus *Gaudeamus* were first reported by Schlosser [18] and mistakenly referred to as *Phiomys andrewsi*; this material, which is of unknown provenance, included two mandibles, one of which preserved teeth in place. Later, Stehlin and Schaub [19] argued that the dentition showed derived features with respect to *Phiomys andrewsi* and suggested that the material represented a new genus (“Genus novem aus dem Fayum”, p. 266). Wood [14] named the genus *Gaudeamus*, and described more material from the early Oligocene Quarry E that he assigned to a new species, *Gaudeamus aegyptius*. The only known records of *Gaudeamus* outside of Egypt are from the early Oligocene Thaytiniti locality, in the Shizar Member of the Ashawq Formation in the Dhofar province, Oman (a single third upper molar) [20], and from the early Oligocene Zallah locality in Libya, which has recently yielded a new and highly derived species, *Gaudeamus lavocati*, described by Coster et al. [21]. Interestingly, *Gaudeamus* is conspicuously absent from the recently reported rodent fauna of Dor el-Talah, Libya [22], which might be older than L-41; this fauna was reported as being of late middle Eocene age by Jaeger et al. [22], but, based on the occurrence of several derived taxa that are not present in the earliest Priabonian levels of the Fayum area, we consider a mid-Priabonian (middle late Eocene) age for the Dor el-Talah rodents to be more likely.

The systematic position of *Gaudeamus* has been a matter of debate. Wood [14] placed *Gaudeamus* in the family Phiomyidae, but noted that “*Gaudeamus*…is so distinct that it probably belongs in another subfamily” (p. 80), and argued that the genus is more closely related to the genus *Thryonomys* (the extant cane rat of sub-Saharan Africa) than to other Fayum rodents. Lavocat [23] and Antoñanzas et al. [24] placed *Gaudeamus* within the family Thryonomyidae; the cladistic analysis presented in the latter study supported Wood's [14] phylogenetic conclusions by recovering a *Gaudeamus*-*Thryonomys* clade to the exclusion of all other Oligocene-to-Recent species, thus implying a ∼33 Ma ghost lineage for *Thryonomys*. Parsimony analyses undertaken by Sallam et al. [16] found *Gaudeamus* to be either nested within Caviomorpha (based on unconstrained parsimony analysis of morphological characters alone), or the sister group of Hystricidae, when analyses were constrained by a molecular scaffold and/or a chronobiogeographic character. Most recently, Coster et al. [21] presented a very similar parsimony analysis to that of Sallam et al. [16], and argued that *Gaudeamus* might be an African caviomorph.

Here we present new fossil evidence from Quarry L-41 that helps to illuminate the origin of the genus *Gaudeamus*. This new material provides the basis for the erection of a new higher taxon, Gaudeamuridae — the first new family of Fayum rodents to be named in over half a century. In addition, two new species of *Gaudeamus* are documented here by nearly complete crania and almost complete upper and lower dentitions. The new species represent the oldest records of the genus *Gaudeamus* to date. More fragmentary material of *Gaudeamus* from L-41 was described in the unpublished Ph.D. dissertation of Holroyd [15], and one species that she named in that work, *Gaudeamus hylaeus*, was used in a subsequent publication [17] despite not yet having been adequately described and figured according to the requirements of the International Code of Zoological Nomenclature (ICZN); this species has since been listed as a *nomen nudum* by Pickford et al. [25]. A further complication is that we recognize an additional new species, *Gaudeamus aslius*, within Holroyd's *G. hylaeus* hypodigm. In order to prevent further confusion in the literature, herein we recognize *Gaudeamus hylaeus* — though as a less variable species than that recognized by Holroyd [15] — but, importantly, provide the first description of the species that unequivocally establishes it as valid given the requirements of the ICZN. This material is described and compared with older, sympatric, and younger Fayum rodents as well as with some living and fossil taxa that share similarities with *Gaudeamus*, and the new species are included in a phylogenetic analysis of living and extinct hystricognathous rodents, building on the previous work of Marivaux et al. [26] and Sallam et al. [16].

## Methods

### Dental terminology and measurements

**Figure 3:**
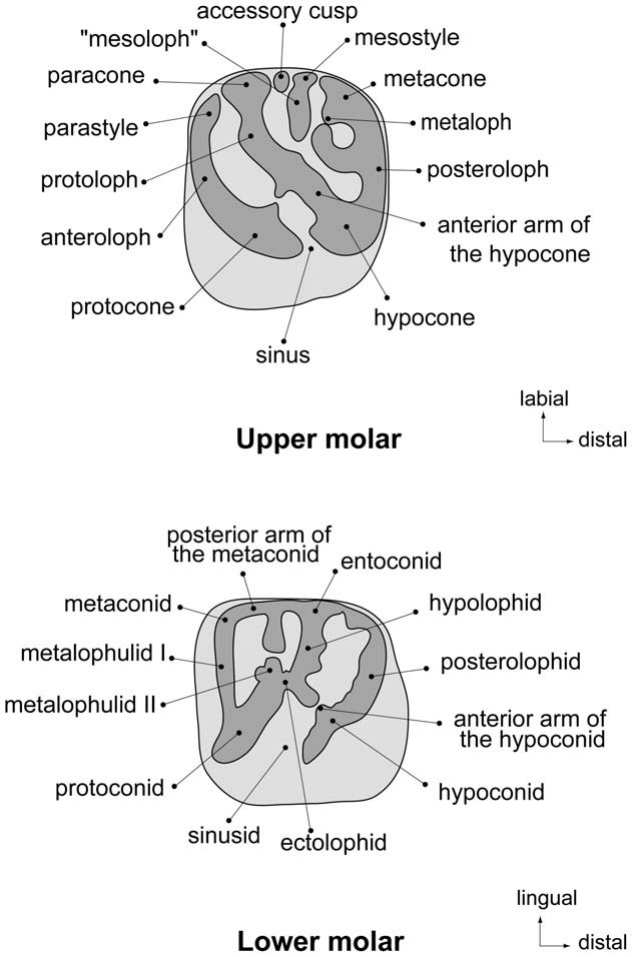
Terminology used to describe features of the second upper and lower molars of *Gaudeamus aslius*, following Marivaux et al. [**26**].

Dental terminology (Figure 3) follows that of Marivaux et al. [26], and the terms “crest” and “loph” are used interchangeably. Teeth are referred to as I, P, and M (for incisors, premolars, and molars, respectively), with upper and lower teeth designated by superscript and subscript numbers (respectively) for locus (e.g., the second lower molar is referred to as M_2_). All dental measurements were taken using a micrometer mounted in the lens of a Meiji binocular microscope. Upper and lower dentitions were whitened using ammonium chloride (NH_4_Cl) in order to produce the figures presented here.

### Phylogenetic analysis

*Gaudeamus*' phylogenetic position was estimated through parsimony analysis of morphological characters, which was undertaken using the heuristic search algorithm in PAUP 4.0b10 [27] across 5000 replicates with random addition sequence and TBR (tree bisection and reconnection) branch swapping. Some multistate characters were treated as ordered and scaled, and in all analyses polymorphisms were assigned a discrete intermediate state. The morphological character matrix (Appendices S1 and S2) builds on the previous work of Marivaux et al. [26] and Sallam et al. [16], and includes 53 living and extinct taxa and 118 morphological characters, almost all of which are from the dentition. The late early Eocene Asian outgroup taxa *Birbalomys* and *Chapattimys* were employed as outgroups.

### Institutional abbreviations

CGM, Egyptian Geological Museum, Maadi, Cairo; DPC, Division of Fossil Primates, Duke Lemur Center, Durham, North Carolina, U.S.A.; LACM, Natural History Museum of Los Angeles County, Los Angeles, California, U.S.A.; MCZ, Museum of Comparative Zoology, Cambridge, Massachusetts, U.S.A.; SBU, Stony Brook University, Stony Brook, New York, U.S.A.

### Nomenclatural acts

The electronic version of this document does not represent a published work according to the International Code of Zoological Nomenclature (ICZN), and hence the nomenclatural acts contained in the electronic version are not available under that Code from the electronic edition. Therefore, a separate edition of this document was produced by a method that assures numerous identical and durable copies, and those copies were simultaneously obtainable (from the publication date noted on the first page of this article) for the purpose of providing a public and permanent scientific record, in accordance with Article 8.1 of the Code. The separate print-only edition is available on request from PLoS by sending a request to PLoS ONE, Public Library of Science, 1160 Battery Street, Suite 100, San Francisco, CA 94111, USA along with a check for $10 (to cover printing and postage) payable to “Public Library of Science”.

In addition, this published work and the nomenclatural acts it contains have been registered in ZooBank , the proposed online registration system for the ICZN. The ZooBank LSIDs (Life Science Identifiers) can be resolved and the associated information viewed through any standard web browser by appending the LSID to the prefix “http://zoobank.org/”. The LSID for this publication is: urn:lsid:zoobank.org:pub:E562334A-95DF-48EB-8928-36562CECAB43.

## Results

### Systematic paleontology

#### Systematic hierarchy

Mammalia Linnaeus, 1758; Rodentia Bowdich, 1821; Hystricognathi Tullberg, 1899.

### Family Gaudeamuridae, new family

#### Diagnosis

As for the genus.

### *Gaudeamus* Wood, 1968

#### Type species

*Gaudeamus aegyptius* Wood, 1968.

#### Included species

*G. aegyptius*, *G. lavocati*, *G. aslius* sp. nov., and *G. hylaeus* sp. nov.

#### Distribution

Late Eocene and early Oligocene of Egypt; early Oligocene of Libya and Oman.

#### Emended diagnosis

Rodents with a well-developed hystricomorphous infraorbital foramen, a hystricognathous mandible, a high coronoid process, and a well-developed postorbital process. Molars are lophodont and unilaterally hypsodont, with thick crests and crestiform but recognizable cusps (i.e., lophs meet cusps at their apices, and hypoconids and hypocones are slightly tilted and internally placed on the crown). The dP^4^/_4_ are replaced by permanent P^4^/_4_. On the lower teeth, the anterior arm of the hypoconid is weakly-developed or absent; the anterocingulid is absent; the hypolophid is oblique, being mesiolabially-to-distolingually oriented; lower molars lack a complete ectolophid (i.e., a connection between the protoconid and hypoconid); P_4_-M_3_ lack the hypoconulid, and dP_4_ is tetralophodont with a well-developed anterolophid. On the upper teeth, the connection between the protoloph and the protocone is either very low and short, or absent; the metaloph varies from being poorly-developed to absent; the protoloph is distolingually oriented and merged into the anterior arm of the hypocone, forming the diagonal crest that divides the crown into two major parts; the mesostyle and central loph (possibly a mesoloph, and referred to hereafter as a “mesoloph” to reflect uncertainty as to whether the crest is best interpreted as a mesoloph or mesolophule) are well-developed; the hypocone is slightly anteriorly tilted on the crown and is placed distal to the protocone on M^1^ and M^2^. The P^4^ has a robust endoloph.

### *Gaudeamus aslius*, sp. nov

urn:lsid:zoobank.org:act:2E7172BD-2EF7-4D4D-B861-26A94BE00115

Figures 4, 5, 6, 7, 8A, 9, 10, 11, 12, 13, Table 1

**Figure 4:**
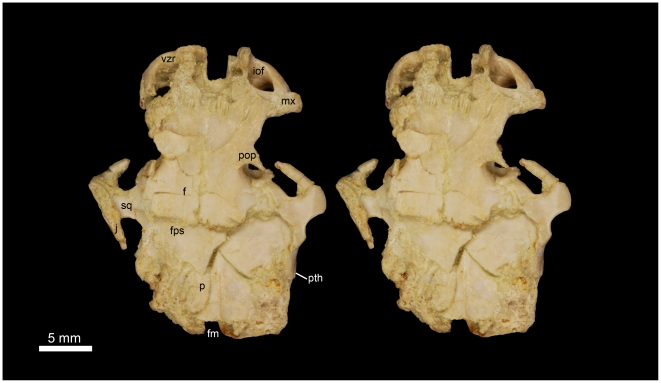
Stereomicrograph of the dorsal view of the holotype cranium elements of *Gaudeamus aslius*, new species (CGM 66006), showing the anatomical features mentioned in the text. Abbreviations: f, frontal; fm, foramen magnum; fps, frontal-parietal suture; iof, infraorbital foramen; j, jugal; mx, maxilla; nfs, nasail-frontal suture; p, parietal; pop, postorbital process; pth, post-tympanic hook; s, squamosal; vzr, ventral zygomatic ramus.

**Figure 5:**
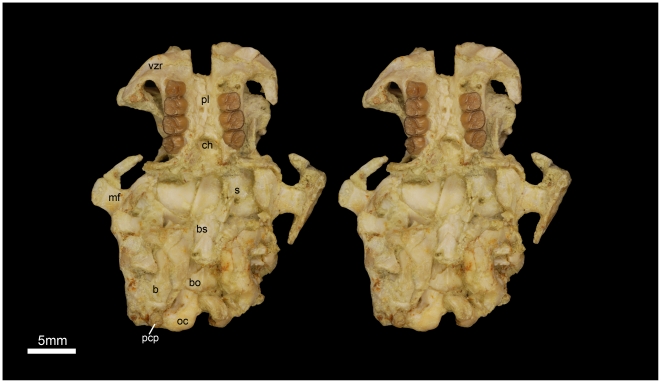
Stereomicrograph of the ventral view of the holotype cranium of *Gaudeamus aslius*, new species (CGM 66006), showing the anatomical features mentioned in the text. Abbreviations: b, bulla; bo, basioccipital; bs, basisphenoid; ch, choanae; mf, mandibular fossa; oc, occipital condyles; pcp, paracondylar; pl, Palate; s, sphenoid; vzr, ventral zygomatic ramus.

**Figure 6:**
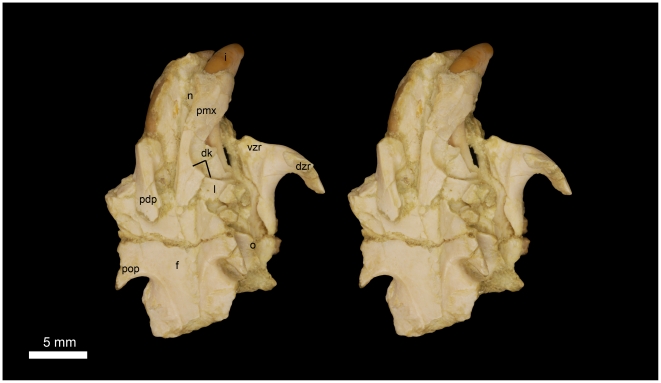
Stereomicrograph of the dorsal view of DPC 16539, *Gaudeamus aslius*, new species, showing the anatomical features mentioned in the text. Abbreviations: dk, dorsal bony keel; dzr, dorsal zygomatic ramus; f, frontal; i, incisor; l, lacremal; n, nasal; o, orbit; pmx, premaxilla; pdp, posterodorsal process; pop, postorbital process; vzr, ventral zygomatic ramus.

**Figure 7:**
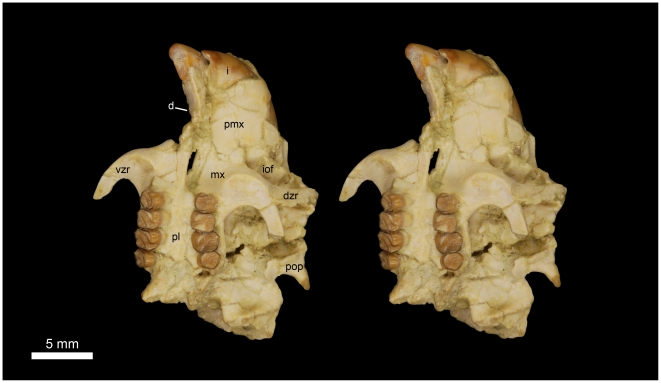
Stereomicrograph of the ventral view of DPC 16539, showing cranial elements of *Gaudeamus aslius*, new species, and the anatomical features mentioned in the text. Abbreviations: d, diastema; dzr, dorsal zygomatic ramus; i, incisor; iof, infraorbital foramen; mx, maxilla; pl, palate; pmx, premaxilla; pop, postorbital process; vzr, ventral zygomatic ramus.

**Figure 8:**
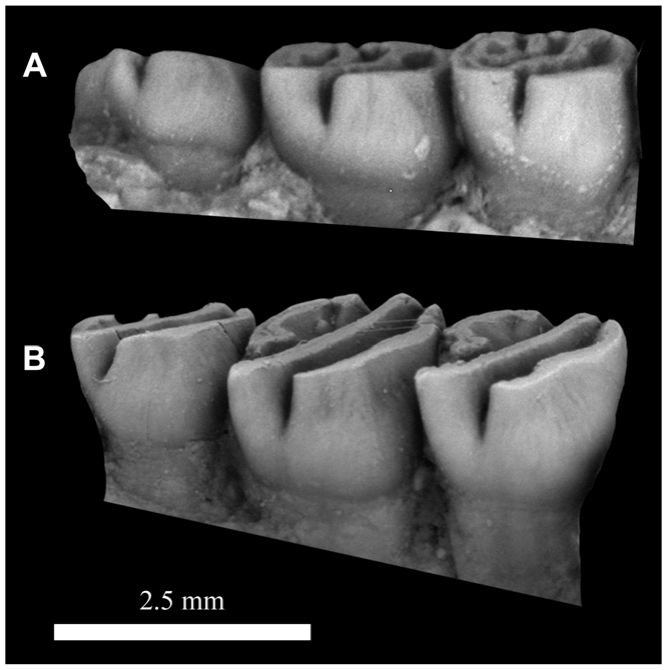
Lingual view of the right upper molars of: A, *Gaudeamus aslius*, new species, CGM 66006; B, *Gaudeamus hylaeus*, new species, CGM 66007, showing the degree to which lingual hyposodonty is expressed in each species.

**Figure 9:**
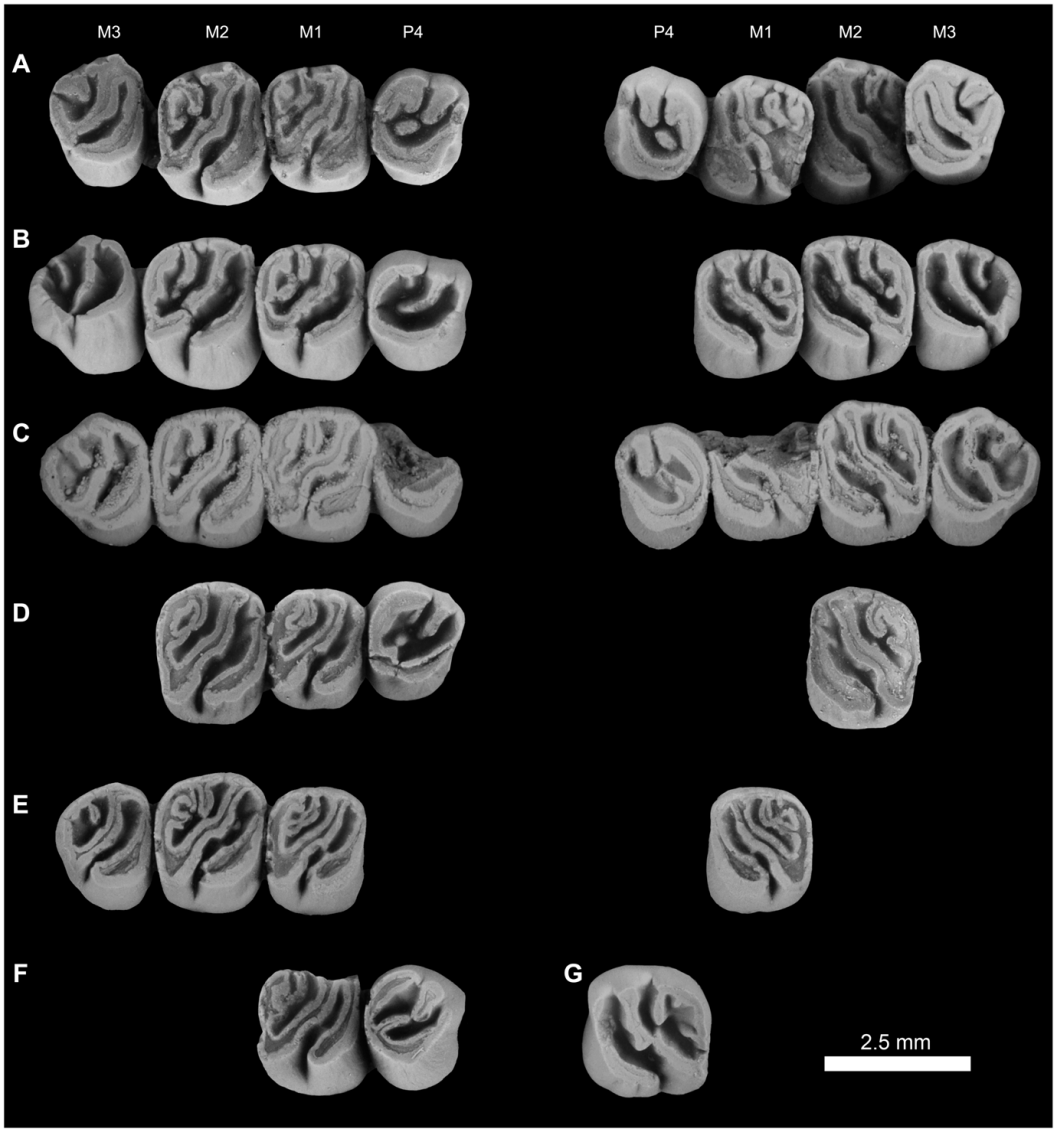
Upper dentitions of *Gaudeamus aslius*, new species. **A**, DPC 16539, right P^4^-M^3^, left P^4^-M^3^; **B**, CGM 66006, right P^4^-M^3^, left M^1^-M^3^; **C**, DPC 20381, right P^4^-M^2^, left M^2^; **D**, DPC 14426, right M^1^-M^3^, left M^1^; **E**, DPC 15239, right P^4^-M^1^ (M^1^ broken); **F**, DPC 13196, right M^1^, reversed. The apparent differences between the two rows in the figure are due to the postmortem distortion and displacement of the crowns.

**Figure 10:**
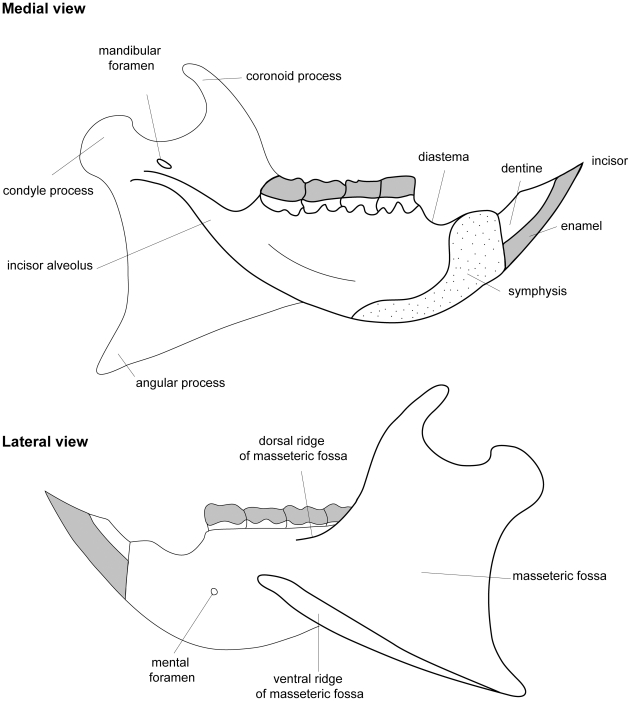
Reconstruction of the mandible of the genus *Gaudeamus*. The restoration is based on combined information from specimens DPC 9456 (corpus and incisor), DPC 12990 (angular process) and DPC15577 (coronoid process).

**Figure 11:**
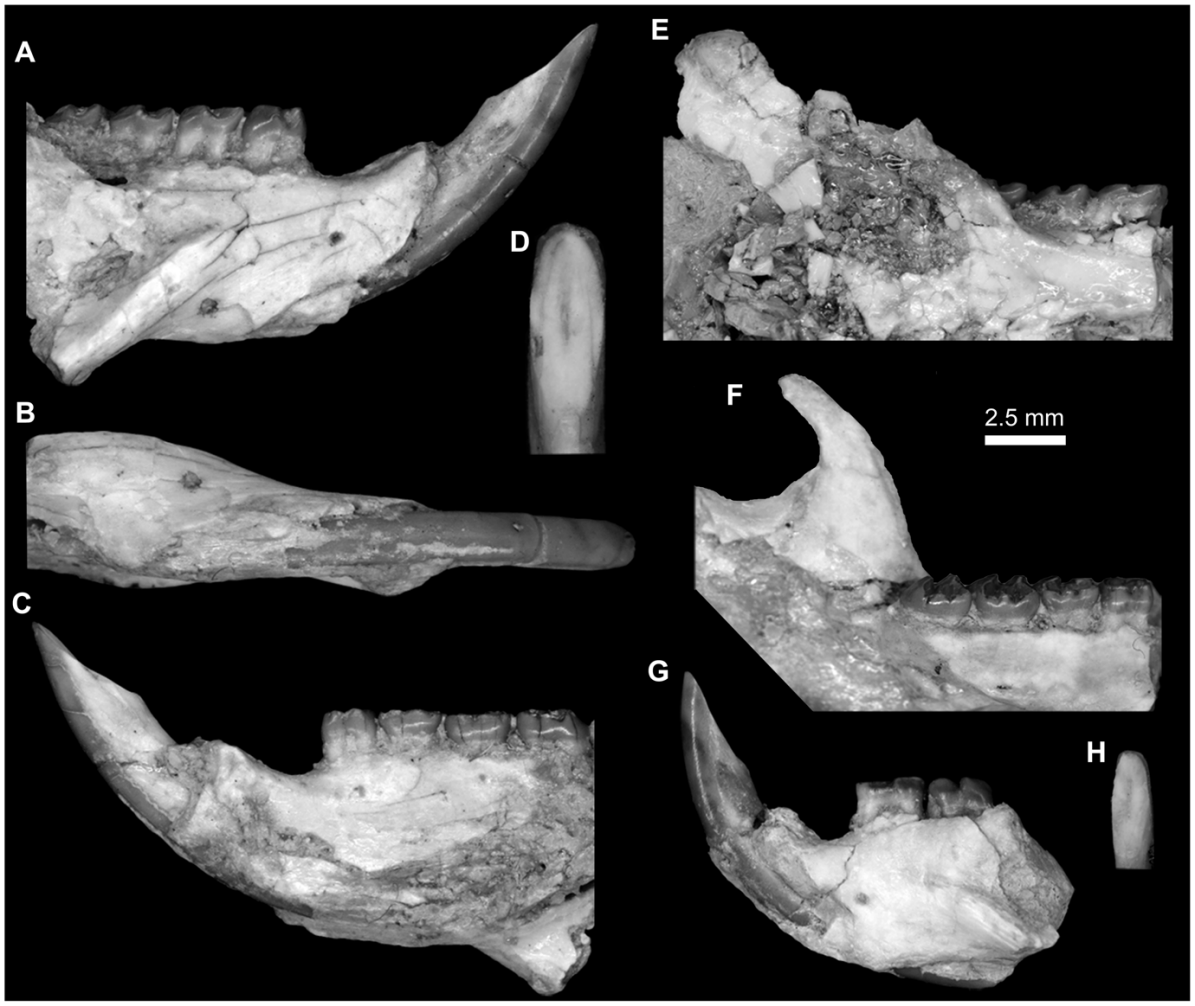
Mandibular fragments and lower dentition of *Gaudeamus aslius*, new species. **A–D,** DPC 17677, fragment of right mandible with P_4_-M_3_ and dislocated incisor. **A**, lateral view; **B**, ventral view; **C,** medial view; **D,** close up occlusal view of incisor; **E,** DPC 20513, fragment of right mandible with well preserved condyle process and M_1–3_; **F,** DPC 15577, fragment of left mandible with well preserved coronoid process and P_4_-M_3_. **G–H,** DPC 17653, fragment of right mandible of a juvenile with dP_4_-M_1_ and displaced incisor. **G**, lateral view, reversed; **H,** close up in occlusal view of incisor. Incisors are displaced due to postdepositional distortion.

**Figure 12:**
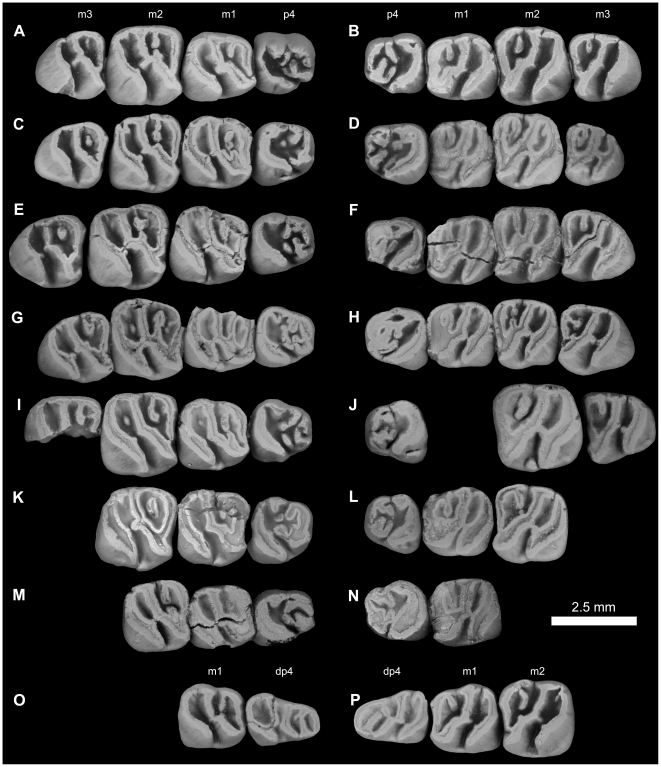
Lower dentitions of *Gaudeamus aslius*, new species. **A**, DPC 20331, right P_4_-M_3_; **B**, DPC 15577, left P_4_-M_3_; **C**, DPC 17677, right P_4_-M_3_; **D**, DPC 15199, left P_4_-M_3_; **E**, DPC 13823, right P_4_-M_3_; **F**, DPC 14413, left P_4_-M_3_; **G**, DPC 11565, right P_4_-M_3_; **H**, DPC 20178, right P_4_-M_3_; **I**, DPC 15526, right P_4_-M_3_ (M_3_ is broken); **J**, DPC 7972, left P_4_-M_3_ (missing M_1_); **K**, DPC 21301, right P_4_ –M_2_; **L**, DPC 16950, left P_4_ –M_2_; **M**, DPC 20457, right P_4_ –M_2_; **N**, DPC 15663, right P_4_ –M_1_; **O**, DPC 17653, right dP_4_ –M_1_; **K**, DPC 16920, right dP_4_ –M_2_, reversed.

**Figure 13:**
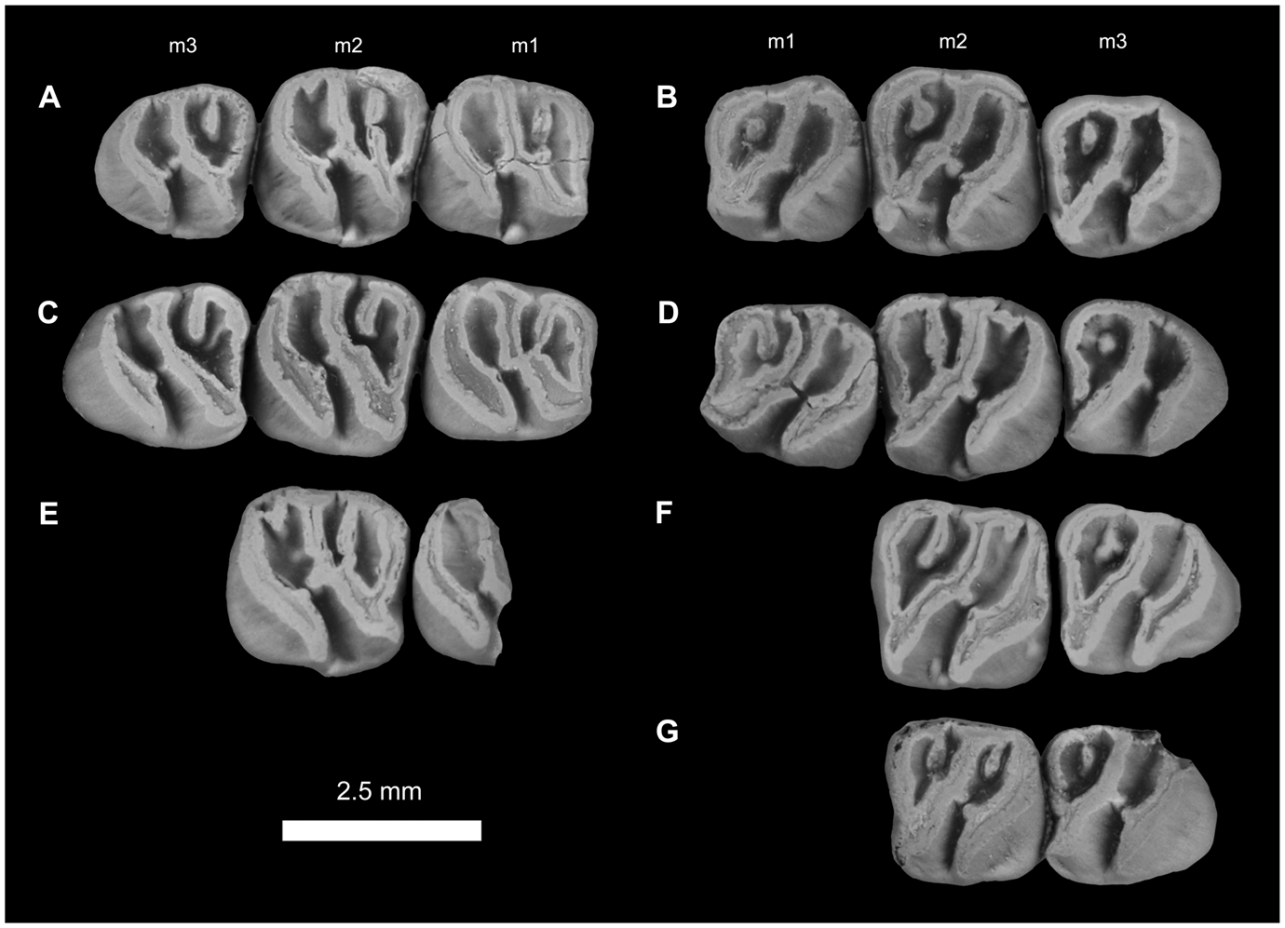
Lower dentitions of *Gaudeamus aslius*, new species. **A**, DPC 16550, right M_1–3_; **B**, DPC 16627, left M_1–3_; **C**, DPC 20513, right M_1–3_; **D**, DPC 8196, left M_1–3_, reversed; **E**, DPC 9453, right M_1–2_; **F**, DPC 17632, left M_2–3_; **G**, DPC 8230, right M_2–3_, reversed.

**Table 1:** Dental metrics for *Gaudeamus aslius*, sp. nov., and *Gaudeamus* aff. *aslius*, in millimeters.

*Gaudeamus aslius*											
		dP_4_		P_4_		M_1_		M_2_		M_3_	
Specimen	side	L	W	L	W	L	W	L	W	L	W
DPC 7972	left	-	-	1.88	1.85	-	-	2.38	2.38	2.13	2.00
DPC 8230	right	-	-	-	-	-	-	2.00	2.05	2.05	1.95
DPC 9453	right	-	-	-	-	-	2.00	2.28	2.13	-	-
DPC 11565	right	-	-	1.83	1.78	2.20	-	2.20	2.33	2.13	2.00
DPC 13823	right	-	-	1.90	1.78	2.25	2.20	2.28	2.50	2.15	2.08
DPC 14413	left	-	-	1.75	1.68	2.08	2.08	2.08	2.28	2.08	2.13
DPC 15199	left	-	-	1.88	1.75	1.98	2.00	2.15	2.25	1.98	1.85
DPC 15526	right	-	-	1.88	1.75	2.10	2.13	2.25	2.45	-	-
DPC 15577	left	-	-	1.88	1.78	2.15	1.98	2.25	2.30	2.05	2.00
DPC 15663	right	-	-	1.83	1.75	2.00	1.90	-	-	-	-
DPC 16627	left	-	-	-	-	2.10	2.00	2.18	2.28	2.25	1.95
DPC 16950	left	-	-	1.65	1.80	2.13	2.05	2.18	2.33	-	-
DPC 17632	left	-	-	-	-	-	-	2.38	2.33	2.38	2.13
DPC 17653	right	2.23	1.50	-	-	2.05	1.88	-	-	-	-
DPC 17677	right	-	-	1.85	1.78	2.13	2.08	2.10	2.28	2.08	1.88
DPC 20178	right	-	-	1.85	1.85	2.00	1.90	1.93	2.08	2.13	2.08
DPC 20457	right	-	-	1.75	1.78	1.98	1.95	1.88	2.13	-	-
DPC 20513	right	-	-	-	-	2.10	1.95	2.08	2.20	2.25	2.03
DPC 21301	right	-	-	1.88	1.88	2.05	2.18	2.23	2.40	-	-
DPC 20331	right	-	-	1.90	1.90	2.25	2.05	2.28	2.35	2.08	2.05
DPC 16920	right	2.23	1.75	-	-	2.15	2.08	2.20	2.38	-	-
DPC 8196	left	-	-	-	-	2.13	2.00	2.25	2.18	2.03	1.88
DPC 16550	right										

		dP^4^		P^4^		M^1^		M^2^		M^3^	
DPC 16539	left	-	-	1.63	2.10	1.80	2.30	-	2.55	1.68	2.23
	right	-	-	1.63	2.13	1.80	2.25	1.95	2.50	1.70	2.25
CGM 66006	left	-	-	-	-	1.88	2.30	2.00	2.58	1.93	2.38
	right	-	-	1.80	2.18	1.88	2.38	2.00	2.55	1.85	2.33
08-207	left			1.58	2.01	-	-	1.95	2.45	1.93	2.23
	right			-	-	1.95	2.33	1.93	2.43	1.88	2.18
DPC 20381	left	-	-	-	-		-	1.93	2.50	-	-
	right	-	-	1.75	2.13	1.75	2.13	1.88	2.50	-	-
DPC 14426	left	-	-	-	-	2.00	2.38	-	-	-	-
	right	-	-	-	-	2.00	2.38	2.15	2.88	1.88	2.50
DPC 15239		-	-	1.83	2.00	1.95	2.18e	-	-	-	-
DPC 13196B	right	2.18	2.65	-	-					-	-

*Gaudeamus aff. aslius*											
		dP_4_		P_4_		M_1_		M_2_		M_3_	
DPC 12990	left	-	-	2.10	1.78	2.03	1.73	2.28	2.18	2.15	2.00

Estimates are indicated by an “e”.

#### Etymology

From Arabic *asl* for origin, in reference to the primitive features of the species with respect to other *Gaudeamus* species.

#### Holotype

CGM 66006, distorted cranium that preserves most elements aside from the snout (premaxilla and nasal bones) and the left P^4^ (Figures 4, 5, 11B).

#### Type locality

Locality 41, lower sequence of the Jebel Qatrani Formation, Fayum Depression, Egypt.

#### Referred specimens

In addition to the holotype, the *Gaudeamus aslius* hypodigm includes two skull fragments, 3 maxillary fragments, and 23 mandibular fragments: DPC 16539, cranial fragments with right and left P^4^-M^3^; 08-207, cranial fragments with right and left P^4^-M^3^; DPC 20381, maxillary fragments with right P^4^-M^2^ and left M^2^; DPC 14426, maxillary fragments with right M^1^-M^3^ and left M^1^; DPC 15239, right maxillary fragment with P^4^-M^1^; DPC 13196B, right isolated P^4?^; DPC 20331, right mandibular fragment with P_4_-M_3_ and broken incisor; DPC 15577, left mandibular fragment with P_4_-M_3_ and complete coronoid process; DPC 17677, right mandibular fragment with P_4_-M_3_ and complete incisor; DPC 15199, left mandibular fragment with P_4_-M_3_; DPC 13823, right mandibular fragment with P_4_-M_3_ and broken incisor; DPC 14413, left mandibular fragment with P_4_-M_3_; DPC 11565, right mandibular fragment with P_4_-M_3_; DPC 20178, right mandibular fragment with P_4_-M_3_; DPC 15526, right mandibular fragment with P_4_-M_3_ and partial incisor; DPC 7972, left mandibular fragment with P_4_ and M_2_-M_3_; DPC 21301, right mandibular fragment with P_4_-M_2_ and complete incisor; DPC 16627, left mandibular fragment with P_4_-M_3_ and complete incisor; DPC 20457, right mandibular fragment with P_4_-M_2_ and broken incisor; DPC 15663, right mandibular fragment with P_4_-M_1_; DPC 17653, right mandibular fragment with dP_4_-M_1_ and complete incisor; DPC 16920, right mandibular fragment with dP_4_-M_2_ and broken incisor; DPC 16550, right mandibular fragment with M_1–3_; DPC 16950, left mandibular fragment with M_1–3_ and complete incisor; DPC 20513, right mandibular fragment with M_1–3_ and complete condylar process; DPC 88.1364, left mandibular fragment with M_1–3_ and complete incisor; DPC 9453, right mandibular fragment with partial M_1_, complete M_2_ and broken incisor; DPC 17632, left mandibular fragment with M_2–3_ and broken incisor; DPC 8230, left mandibular fragment with M_2–3_ and broken incisor.

#### Age and distribution

All specimens are from the terminal Eocene (latest Priabonian) in age (∼34 Ma), Quarry L-41, lower sequence of Jebel Qatrani Formation, Fayum Depression, northern Egypt.

#### Diagnosis

*Gaudeamus aslius* differs from other *Gaudeamus* species in having relatively short upper molars; lophs that are relatively transversely oriented; a more sinuous diagonal crest on the upper molars; a relatively well-developed “mesoloph”; a low connection between the protocone and protoloph; a relatively well-developed anteroloph on P^4^; a relatively well-developed metalophulid I, anterior arm of the hypoconid, and metalophulid II on the lower molars; and in having an ectolophid and accessory cusp on the metalophulid I of P_4_. In addition, *G. aslius* is larger and has more hypsodont teeth than *Gaudeamus aegyptius*, and is less hypsodont than *Gaudeamus hylaeus* sp. nov.

### Description

#### Cranial elements (Figures 4, 5, 6, 7)

Two nearly complete but crushed crania of *Gaudeamus aslius* together document the cranial morphology of *Gaudeamus* for the first time. The holotype cranium CGM 66006 (Figures 4–5) bears most of the cranial elements aside from the snout (premaxillae and nasals) and the left P^4^. The cranium DPC 16539 (Figures 6–7) contains the premaxillae with two large upper incisors, both maxillae with the entire dentition (P^4^-M^3^), and most of the frontal. Both crania represent adult individuals with with worn permanent premolars. The specimens are of roughly similar size. Severe post-mortem distortion has led both crania to be dorsoventrally flattened, and the specimens bear numerous surface cracks and displacements. Some of the cranial elements are also overlapping and/or fused together, which makes it difficult to confidently trace and describe the extent of some bones, particularly in the orbit and around the auditory bulla. DPC 16539 was subjected to a slightly more medio-laterally oriented force post-deposition, leading to asymmetry of the cranial surface. However, together these specimens provide important information about the cranial morphology of *Gaudeamus*.

Because cranial remains of early hystricognaths are extremely rare, the description of the cranial elements is based primarily on comparisons with living taxa — the African phiomorph *Thryonomys* (MCZ 56868), the South American caviomorph *Cavia* (SBU-MRd 29), and the African hystricid *Atherurus* (SBU-MRd 5), supplemented by comparisons with cranial remains of the fossil caviomorphs *Branisamys* and *Incamys* from the Oligocene of Bolivia figured by Wood and Patterson [28]. As with all fossils from L-41, the *Gaudeamus* crania were originally entombed in a pale green hygroscopic claystone matrix, which became fully embedded in sutures, foramina and cracks. Cleaning of the entire matrix is not only a difficult and painstaking task, but also weakens the specimens and makes them vulnerable to breakage.

The rostrum is moderately long with paired nasal bones that can only be seen in DPC 16539 (Figures 6–7). The nasal bones are extremely fragile and highly damaged. They extend backward to articulate with the frontals at the level of P^4^-M^1^, and slightly posterior to the infraorbital foramen. In dorsal view, the suture between the nasal and the premaxilla is not visible.

The premaxillae are preserved in DPC 16539. The bones house two large upper incisors and form most of the rostrum (wall, floor, and upper diastema). The most rostral part of the right premaxilla bears three tiny foramina parallel to the naso-premaxilla line. In dorsal view, the posterodorsal process of the premaxilla is narrow anteriorly, flares posteriorly, and has a flat surface with relatively larger foramina. It is bounded posteriorly by the frontal bone and posterolaterally by the maxilla and, presumably, the lacrimal. The suture between the frontal and premaxilla is located approximately medial to the infraorbital foramen as in *Thryonomys*, and slightly posterior to the frontonasal suture. The medial sides of the posterodorsal processes are parallel.

In lateral view, the anterior portion of the premaxilla is narrower than the posterior part, leading to a curved diastema as in *Thryonomys*. The premaxilla is smooth laterally and has a dorsal bony keel that runs along the lateral side of the posterodorsal process (Figure 6) as in *Thryonomys* and *Atherurus*. This ridge occupies the dorsal border of the masseteric fossa and serves as the origin of the deep masseter muscle that passes through the infraorbital foramen. At the anterior end of this keel, there is an elevation on the middle of the lateral wall of the premaxilla; this could be the site of origin of the medial masseter as in *Atherurus*. The incisive foramina are obscured due to distortion. The ventral region of the premaxilla forms almost two-thirds of the upper diastema, a similarity to *Thryonomys* and *Cavia* that is not seen in *Atherurus*. The suture between the premaxilla and the maxilla on the lateral surface of the rostrum is not well-preserved, but it arcs anteriorly and continues ventrally, anterior to the infraorbital foramen (Figure 7).

The maxillae contain P^4^-M^3^ in DPC 16539, but the left P^4^ is missing in CGM 66006. In ventral view, the anterior portion of the maxilla extends deep to the plane of the alveoli as occurs in *Thryonomys* and *Cavia*, but not *Atherurus*. Part of the suture for the premaxilla is present in both crania. There are no upper third deciduous premolars in either cranium, however both individuals had already replaced DP^4^ – as such, it is possible that DP^3^ was present and retained for a short time in juveniles. Laterally, the facial process of the maxilla meets the posterior part of the premaxilla to form the lateral wall of the rostrum, and forms the medial portion of the infraorbital foramen. As is typical of ctenohystrican rodents, the infraorbital foramen is hystricomorphous, and the medial masseter muscle passed through the foramen to attach to the premaxilla, as mentioned above. The infraorbital foramen is made up entirely of the maxilla, and lies anterodorsal to the tooth-row. On the right side of the DPC 16539, the infraorbital foramen has a ventrolaterally rounded out line, whereas on the other side, and on the both sides of CGM 66006, the foramen became dorsoventrally compressed postmortem.

The ventral ramus of the zygomatic process of the maxilla, which forms the ventral ridge of the infraorbital foramen, is thin and extends laterally from the area in front of P^4^ and then curves posteriorly, delimiting the anterovental portion of the orbital margin. The anteroventral part of the ventral zygomatic ramus (Figures 5 and 7) bears a deep fossa for the origin of the superficial masseter muscles, and, posteriorly, a relatively shallow fossa for the origin of the lateral masseter, as in *Cavia*. These fossae are weakly developed in *Atherurus*, while the more anterior fossa is replaced by a well-developed tubercle in *Thryonomys*. The dorsal zygomatic ramus is narrow and dorsally oriented as in *Thryonomys*. It is concave posteriorly along its length, which might indicate that the anterior part of the jugal was plate-like and attached to the dorsal zygomatic ramus as in *Thryonomys* and *Atherurus*. The roots of the dorsal and ventral zygomatic rami extend anteriorly to roughly the same point, suggesting that the infraorbital foramen was vertical as in *Thryonomys*, rather than rostroventrally oriented as in *Cavia* and *Atherurus*. The dorsal ramus of the zygomatic arch continues anteriorly with the lateral keel of the posterodorsal process as in *Thryonomys* and *Atherurus*. In CGM 66006, the infraorbital foramina appear to be tilted posteriorly due to compression. On the left side of CGM 66006, there is an incompletely preserved jugal that is displaced backward and situated lateral to the mandibular fossa. On both sides of CGM 66006, the posterior part of the zygomatic arch is slender and extends anteriorly from the squamosal to end as a thin splint, which indicates that the jugal probably tapered posteriorly. The shape and height of the jugal, and its contact with the lacrimal bone, are uncertain.

The original morphology of the palate is difficult to determine due to distortion, but generally appears to be somewhat flat, slightly lower than the alveolar plane, and broad throughout its length as in *Thryonomys and Atherurus*, and not narrow anteriorly as in *Cavia*. It houses the major palatine foramina, which are relatively round and large, and which lie at the level of the first upper molar as in *Thryonomys* and *Atherurus*. In DPC 16539, only the right half of the palate is exposed, as the left half has been displaced dorsally.

As in *Thryonomys* and *Cavia*, the infraorbital fissure is relatively broad, forming the floor of the orbit and separating the orbital process from the alveolar portion, but *Gaudeamus* differs from those taxa in having a relatively low orbital process. *Gaudeamus*, *Cavia* and *Thryonomys* differ from *Atherurus* in having both the infraorbital fissure and the orbital process. On the medial wall of the right infraorbital fissure of DPC 16539, a small alveolar foramen is exposed. None of the other orbital foramina can be identified due to damage to the orbital wall.

The outline and contacts of the lacrimal bone are uncertain due to distortion. However, the suture between the posterodorsal process of the premaxilla, and that with the dorsal zygomatic ramus is preserved on the right side of DPC 16539, and it appears that the lacrimal occupies the dorsal and posterior aspect of the dorsal zygomatic ramus. There is a small foramen on the dorsal surface of the lacrimal that is preserved on both sides of DPC 16539, but is not obvious on CGM 66006. The lacrimal foramen is obscured.

The paired frontal bones together occupy the middle third of the length of the cranium and are highly fractured. In DPC 16539, the most posterior portion of the frontals is broken, but is mostly complete in CGM 66006. Dorsally, the frontals are roughly rectangular and slightly flat, with a shallow longitudinal curve toward the interfrontal suture and become narrow at its middle as in *Atherurus*. An interfrontal suture separates the frontals along their length, which is a feature found in the living and extinct caviomorphs and phiomorphs [29].

A striking character of the *Gaudeamus* crania is a triangular and relatively large postorbital process, which extends laterally from the middle part of the frontal. This process is much smaller in the other living and extinct hystricognathous rodents in our comparative set. There is a slight postorbital constriction posterior to the process, forming the narrowest point of the frontals. The temporal line curves posteromedially from the postorbital process. The temporal lines are faint in CGM 66006, but distinct in DPC 16539, perhaps due to more advanced age of the latter as suggested by the more worn molars of that specimen. On DPC 16539, there is a depression at the posterior part of the frontals, medial to the temporal lines, which presumably where the squamosal overlaps the frontals, as in *Atherurus*. The frontals meet the parietals at a level somewhat anterior to the bullae, forming a straight frontoparietal suture, best seen in dorsal view on CGM 66006.

The parietals are preserved on CGM 66006, but missing on DPC 16539. They are slightly arched laterally, and form the dorsocaudal third of the cranium. The parietals are badly crushed and cracked, and its anterior part has been displaced underneath the frontals, but the coronal suture is preserved on the frontals and is transversely oriented as mentioned above. The interparietal suture cannot be observed. The temporal lines converge into a low sagittal crest at the midpoint of the parietal; the crest increases in height posteriorly. The morphology of the sagittal crest is more similar to that of *Atherurus* than to that of *Thryonomys* or *Cavia*. There is no hint of interparietal separation. The parietals extend back to the level of the foramen magnum. The articulation with the occipital bone is not preserved. *Gaudeamus* clearly lacks the foreshortened, mediolaterally broad, transversely plate-like, and anteriorly inclined configuration of the occiput seen in early Miocene *Bathyergoides* from East Africa [30] and early Oligocene *Tsaganomys* from Mongolia [31]. Laterally, the parietals are bounded by a post-tympanic hook, which is broken and only seen in the right side of CGM 66006.

The alisphenoid is partially preserved, reduced in height as in later hystricognaths [29] and protrudes ventrally from the lateral side of the basisphenoid. In ventral view, the pterygoid fossa is U-shaped, but the pterygoid processes are displaced and badly damaged on both crania, making it difficult to distinguish the medial and lateral pterygoid processes. The choanae are large and open at the level of M^3^ as in *Thryonomys* and *Atherurus*. In *Cavia* and *Incamys*, the choanae are more anteriorly placed, at the level of M^2^. In CGM 66006, the posterior border of the palate is slightly displaced anteriorly due to distortion. The basipharyngeal canal is completely obscured by a mixture of matrix and delicate bone fragments.

The body of the squamosal forms the caudal part of the orbital rim, and sends a sliver of bone anteriorly to contribute to the caudal part of the zygomatic arch. Both mandibular fossae are preserved on CGM 66006; they are elongate and bordered laterally and medially by longitudinal ridges. The post-tympanic hook is a flat process that extends caudally from the squamosal bone, but it is uncertain how far backward this hook extended because its most caudal tip is missing.

The sphenoid is completely flattened in CGM 66006. On the left side, medial to the mandibular fossa, are two foramina anterior to the bulla, one of which could represent the foramen ovale. The bullae are preserved in CGM 66006, but are highly fractured due the dorsovental compression. The bullae are presumably composed entirely of the ectotympanic, as in other rodents, and occupy about a fourth of the entire length of the cranium and are slightly larger relative to cranial length than those of *Atherurus*, *Cavia*, and *Thryonomys*. Their ventral surfaces are strongly arched longitudinally and transversally. The petrosal, and other aspects of middle ear morphology, are completely obscured by the bullae.

Between the two bullae, the basioccipital and the basisphenoid are partially preserved. The latter's caudal portion flares posteriorly and overlaps the basioccipital. On the lateral sides of the basisphenoid, there are two depressions in the position of the foramen lacerum. The most caudal part of the basioccipital is exposed due to crushing of adjacent bones, and is characterized by a faint median keel that runs along its entire length. The right jugular foramen is exposed on CGM 66006, is slit-shaped, and situated at the caudal end of the bulla. The supraoccipital and exoccipital bones are badly damaged. However, the occipital condyles and the right paracondylar process are preserved. The occipital condyles are large, elongate, and thick, and border a large foramen magnum at the ventral aspect of the occipital bone, as in *Thryonomys* and *Atherurus*. On the lateral side of the right condyle, the condyloid canal is preserved. The paracondylar process is short and projects ventrally lateral and caudal to the foramen magnum and bulla, respectively. It is uncertain how far ventrally the paracondylar process might have projected. The condyloid notch is preserved, but displaced.

The two upper incisors of *Gaudeamus aslius* are preserved only in DPC 16539 (Figures 6 and 7). The upper incisor is relatively short and curved when compared with the lower incisors. The upper incisor is oval in outline (mesiodistal length = 1.82 mm, buccolingual length = 3.57 mm) with a flat medial surface and curved mesial and distal surfaces, and has a smooth surface as in *Atherurus* and *Cavia*, but in *Thryonomys* the upper incisor has a somewhat triangular occlusal surface and thick striations on the mesial surface. Enamel covers the mesial surface and extends across about one-third of the lateral side but not as far on the medial side, as in *Atherurus*. In lateral view, the occlusal surface is deeper when compared with that of the lower incisor. The pulp cavity is short and slit-shaped. On the lateral surface of the right premaxilla, the posterior end of the upper incisor is exposed, revealing that the tooth terminates in front of the tooth row as in *Cavia*. In *Thryonomys*, the posterior end of the incisor terminates dorsal to P^4^-M^1^ [32], but in *Atherurus* the posterior end of the incisor terminates roughly in the middle of the diastema.

#### Upper dentition

The upper cheek teeth have unilaterally hypsodont crowns, with the lingual side higher than the labial side (Figure 8A). All cusps and lophs are, from the occlusal view, at the same level, but the cusps are easily recognizable. Within the tooth row, the size increases from P^4^ to M^2^, but M^3^ is slightly smaller than M^1^.

The P^4^ has a roughly oval outline and is broad relative to length. The tooth is smaller than M^1^ and bears four primary cusps (paracone, metacone, protocone and hypocone). The protocone is a large and crestiform cusp that is mesiolabially oriented, forming most of the mesiolingual border of the tooth. The anteroloph is gracile but high, and sits at the same level as the protocone apex; it extends from the mesiolabial side of the protocone to contact the base of the mesial side of the paracone, from which it is separated by a narrow and shallow notch. The paracone is a well-developed and isolated cusp that is placed transverse to the protocone. The labial part of a robust protoloph runs distolingually from the paracone. In CGM 66006 (CGM 66006, Figure 9B), the incomplete protoloph curves toward the posterior margin of the tooth, but it does not reach the posteroloph. There is a variably developed crest and/or swelling between the lingual end of the protoloph and the hypocone, which could be interpreted as either an extension of the protoloph, an interrupted anterior arm of the hypocone, or even a disjunct extension of the metaloph. On the holotype, this swelling is absent. In specimen 08-207, the latter connects to the hypocone, forming a long anterior arm of that cusp. The hypocone is somewhat smaller than the protocone and situated distal to it, and the two cusps are connected by a robust and tall endoloph. The protoloph and the aforementioned crest divide the P^4^ basin into two roughly equal parts. The metacone is a robust cusp that is separated from the paracone by a shallow and narrow notch. The metacone bears a short lingually-oriented metaloph on some specimens, while on others the metaloph is absent. A posteroloph courses around the distal margin of the crown. The posterior basin is only open labially via the narrow notch between the paracone and metacone.

The M^1^ is somewhat rectangular in outline. The crown has four main cusps and a robust mesostyle, all of which are integrated into an incipiently tetralophodont occlusal pattern. The anteroloph is high and robust and courses across the mesial margin of the tooth. It extends mesiolabially from the crestiform protocone and turns labially at its midpoint, ultimately fusing with a well-developed parastyle mesial to the paracone. The parastyle and paracone are separated by a narrow notch. In some specimens (CGM 66006, DPC 13196B and DPC 14426), this notch does not extend down to the level of the anterior basin. The protocone is well-developed and is roughly the same size as the hypocone. The two cusps are separated from each other by a narrow and deep sinus. In a few specimens that show appreciable wear, the protocone is connected to the lingual side of the protoloph via a low crest; this is a remnant of the primitive occlusal pattern seen in more generalized hystricognaths from the Fayum succession. The protocone extends distally as a small projection, but it does not reach the mesial portion of the hypocone (i.e., there is no endoloph). In relatively unworn teeth, the anterior basin appears to be continuous with the lingual sinus, forming an elongate sinuous basin that is open lingually (i.e., the teeth bear the “taeniodont” pattern). The tooth bears a trenchant crest that runs diagonally (mesiolabially to distolingually) across the crown, dividing it into two major parts. This crest is a combination of the protoloph, the anterior arm of the hypocone, and, judging from the step-like midpoint that is evident in some individuals, the mure as well. The protoloph therefore runs distolingually from the paracone, rather than transversely as in other Fayum hystricognaths. The hypocone and its anterior arm are well-developed, forming the lingual part of the diagonal crest. From the distal aspect of the hypocone, a well-developed posteroloph runs labially to fuse to the metacone, forming the posterior margin of the tooth. The metacone is a well-developed cusp and is placed transverse to the hypocone and distal to the paracone. The metaloph is an incipient crest that protrudes from the metacone. There is no hint of a metaconule or mesolophule. In some individuals such as CGM 66006 and DPC 14426, a small metaloph unites with the accessory crest that protrudes from the posteroloph, which together form a small fovea at the distolabial corner of the crown.

The configuration of the labial wall shows considerable variation. In CGM 66006, the labial wall accommodates two small but distinct cuspules, the distal of which is the mesostyle. Three shallow and narrow notches are present on the labial side, but they do not reach the base of the crown (Figure 9B). In DPC 16539, the right M^1^ has a relatively tall labial wall, uninterrupted anterior and posterior arms of the metacone and paracone, respectively, and a tiny mesostyle, while DPC 14426 bears a mesostyle that is centered on the labial wall and there are no notches in the labial wall. The “mesoloph” runs lingually from the mesostyle and curves distally to meet the junction between the metaloph and the accessory crest of the posteroloph, delimiting another fovea. In specimen 08-207, the “mesoloph” is transversely oriented and reaches the diagonal crest.

The M^2^ is the largest upper tooth and has a similar occlusal morphology to that of M^1^, but it is broader and has relatively well-developed lophs, and in some individuals a relatively wide anterior portion. The metaloph and the accessory crest of the posteroloph are also relatively short when compared with those of M^1^. The parastyle is particularly well-developed on DPC 20381. The “mesoloph” is relatively long and oriented either toward the posteroloph or toward the major diagonal crest.

The M^3^ has a relatively triangular outline, with a narrow lingual portion and a broad labial wall. It is the smallest upper molar, and differs from M^1–2^ in having an even more crestiform protocone, which together with the anteroloph sweep around the lingual portion of the tooth, ending just in front of the hypocone. This arrangement leaves the anterior basin open posteriorly, rather than lingually. The metacone is relatively small, does not have a metaloph, and is relatively lingual in position with respect to the mesostyle and the paracone. The hypocone is similarly reduced in size, and relatively labial in position. The major diagonal crest tends to be relatively straight on M^3^. The mesostyle is distinct, and the “mesoloph” is relatively short, never reaching the diagonal crest. The endoloph is either interrupted by a small notch or complete, connecting the protocone and hypocone.

#### Mandible

A complete mandible of *G. aslius* has not been found at L-41, but here we present a composite mandible based on combined information from various well-preserved mandibular fragments in the hypodigm (Figure 10). The mandible is robust, and, as in other hystricognaths, the angular process is placed lateral to the incisor and tooth row, leaving a wide groove between the angular process and the incisor alveolus in ventral view. This groove provides a zone of insertion for the *pars reflexa* of the superficial masseter muscle [33]. The ventral surface outline of the horizontal ramus is convex, with the deepest point being below the P_4_; in lateral view, the ventral masseteric ridge crosses the ventral surface of the horizontal ramus under M_1–2_, and is somewhat similar to *Thryonomys* in this respect. The coronoid process is only known from the specimen DPC 15577 (Figure 11F). Its base is long, and the posteriorly inclined ascending ramus arises lateral to the alveolar plane at the middle of the third molar, leaving a wide groove similar to that of *Atherurus* and *Thryonomys*. The anterior margin of the coronoid process is convex anteriorly, while its posterior margin is concave. The tip of the coronoid process is higher than the condylar process and is pointed distally, forming a distinct hook, as in some bathyergids. The condylar process (Figure 11E) is much higher than the tooth row and the tip of the lower incisor, and has an oval articular surface whose axis is directed anteroposteriorly. The masseteric fossa is relatively deep when compared with those of *Atherurus*, *Cavia*, and *Thryonomys*, and it is broad posteriorly and tapers anteriorly to end beneath the P_4_-M_1_ as in *Thryonomys*. The dorsal masseteric ridge is low and extends anteriorly from the base of the coronoid process and fades below M_1_. The ventral masseteric ridge, which serves as a site of origin for the lateral masseter muscle, is relatively robust, when compared with those of *Atherurus*, *Cavia*, and *Thryonomys*, extends laterally at the midpoint of the corpus, and continues posteroventrally towards the angular process. The posterior terminus of the angular process is sharp and extends posteriorly to the same point as the condylar process, as in *Atherurus*. The mental foramen is small, has a roughly oval outline, and lies under the posterior portion of the diastema as in *Thryonomys*. In young individuals, the mental foramen is placed beneath the trigonid of the dP_4_ (Figure 11G). The diastema is smoothly curved, makes up about half the length of the tooth row, and is shorter than that of the upper jaw. In this respect, *Gaudeamus* is more similar to *Thryonomys* than to *Atherurus* or *Cavia*. On the medial surface of the mandible (Figure 10), the mandibular foramen is oval, and is situated in the area between the coronoid and condylar processes, on the dorsal margin of a strut that extends posteriorly from the rear part of the incisor alveolus, as in *Thryonomys*. The symphysis is distinctly concave along its dorsal surface, with its thickest part anteriorly placed; the symphysis tapers posteriorly to end below the P_4_.

The lower incisor (Figure 11D and H) is oval in cross–section, with a somewhat flat and smooth medial margin and a convex distal margin. The anterior surface of the incisor is covered by smooth enamel that extends onto the lateral and medial surfaces, covering almost half of the lateral side and one-third of the medial side of the incisor. The pulp cavity is elongate in outline and sits in the middle of the dentine layer. The incisor becomes increasingly robust and thick with age (Figure 11D). In many respects, the lower incisor of *Gaudeamus* is more similar to those of *Atherurus* and *Cavia* in than that of *Thryonomys*; the latter genus has a flat mesial surface with a thin layer of enamel that covers only a small part of both sides, and has a triangular occlusal surface with a rounded pulp cavity.

#### Lower dentition

The dP_4_ of *G. aslius* (Figure 12O, P) is only known from two specimens (DPC 16920 & DPC 17653). The dP_4_ is replaced by the permanent P_4_ as in other species of *Gaudeamus*. The tooth is longer than it is wide, and has a triangular outline, with a broad talonid and a narrow trigonid. The occlusal pattern is essentially pentalophodont, with four major cusps (metaconid, entoconid, protoconid, hypoconid) and a weakly developed hypoconulid. The most convex mesial crest is the metalophulid I, which runs from the mesial portion of the protoconid toward the anterior side of the metaconid. The latter cusp is placed transverse to the former. Unlike sympatric and younger Fayum hystricognaths, there is no anterocingulid and no anteroconid on the anterior portion of the tooth. The crest distal to the metalophulid I is the posterior arm of the protoconid ( = metalophulid II). In DPC 17653, the metalophulid II curves distally and then runs transversely to fuse to the labial side of the metaconid, delimiting the anterior basin of the tooth. But in specimen DPC 16920, the metalophulid II is oriented distolingually, toward the mesolophid, but does not attach to that crest. The ectolophid runs from the distal side of the protoconid to fuse to a well-developed cusp (possibly the mesoconid), and continues distolingually toward the hypolophid. A shallow notch interrupts the ectolophid distal to the protoconid. A complete mesolophid represents the third major loph that runs lingually from the distal part of the ectolophid to fuse with a well-developed mesostylid, dividing the middle basin into two roughly equal foveae. The mesostylid is situated midway between the entoconid and the metaconid, and is connected to the metaconid via a trenchant posterior arm of the latter's cusp. The hypolophid is the fourth main crest of the crown, is slightly oblique in orientation, and connects a large entoconid to the junction between the ectolophid and the anterior arm of the hypoconid. In DPC 17653, the connection between the hypolophid and the ectolophid is relatively weak when compared with that of DPC 16920. The posterior division of the middle basin is open lingually, due to the absence of the anterior arm of the entoconid. The posterolophid is the most distal crest on the crown, and runs distolingually from the mesiolabially oriented hypoconid to course around the posterior margin of the tooth. The anterior arm of the hypoconid is well-developed in DPC 17653, but it is interrupted in DPC 16920. The posterior basin is relatively wide when compared with the anterior and middle basins, and is open lingually via a small notch in its lingual wall. The hypoconulid is represented by a minor swelling along the posterolophid, and is barely perceptible on DPC 17653.

The P_4_ (Figure 12) is generally pear-shaped in outline, with a wide talonid and a narrow trigonid. The P_4_ is relatively short and broad when compared with dP_4_ and has four major cusps (metaconid, protoconid, entoconid and hypoconid). In most unworn teeth (Figure 12A), the four major cusps are recognizable, with the mesostylid variably so. The metaconid and the protoconid are transversely placed and are connected via the most mesial crest (metalophulid I), which runs from the mesiolabial side of the metaconid and connects to the mesiolingual portion of the protoconid. In some individuals (DPC 15199 and DPC 20331), a well-developed cusp occurs along the length of the metalophulid I, between the protoconid and metaconid. The metalophulid I is generally interrupted labially by a narrow notch that is lingual to the protoconid, but there is considerable variation in this area: in DPC 15577, the metalophulid I is not interrupted and fuses to the lingual side of the protoconid, and in DPC 13823, the metalophulid I connects to the base of the protoconid's anterior arm, leaving a deep crevice in the mesial wall of the crown. The posterior arm of the protoconid is short, oriented toward the metaconid, and terminates near the midline of the tooth. In DPC 15526, the posterior arm of the protoconid is complete and reaches the metaconid, but is very short in DPC 17677. The hypolophid varies from being absent to incipient. In DPC 15577, the hypolophid runs distally and connects to the posterior wall of the tooth, delimiting a small fovea. In some individuals, there is an accessory cusp that is positioned mesiolabial to the hypolophid. In two individuals (DPC 14413 and 20178), the hypolophid extends mesially to connect with the metaconid, and, together with the lingual wall, delimits a small longitudinal basin. The mesostylid is well-developed, is placed between the metaconid and the entoconid, and has a trenchant accessory crest that extends labially and ends near the midline of the tooth. There is no anterior arm of the entoconid, leaving a narrow notch in the lingual wall, but the posterior arm of the metaconid is generally high and connected with the mesostylid (some individuals, such as DPC 15577, 17677, and 15526 bear small notches in this region). The posterolophid runs distolingually from the crestiform hypoconid and delimits the posterior margin of the tooth, ultimately terminating at the distal aspect of the entoconid, where there is a shallow crevice. The ectolophid is well-developed and incomplete and extends a short distance from the distal side of the protoconid in the direction of the entoconid; it does not connect to the hypoconid as it does more generalized hystricognaths. The hypoconid has a short mesiolabial extension, leading to a narrow labial sinusid.

The M_1_ (Figures 12 and 13) is relatively large and broad when compared with P_4_. The crown has three primary crests that have largely subsumed the cusps. The metalophulid I arises labially from the mesial and labial portion of the metaconid and fuses with the mesiolingual side of the protoconid; it is generally transverse but sometimes curves distally. In relatively unworn specimens (e.g., DPC 17653 and 16600), the metalophulid I connects to the most mesial tip of the protoconid, leading to a narrow and shallow labial extension of the anterior basin. In one specimen (DPC 20331), the metalophulid I and protoconid are separated by a narrow notch. The metaconid is transverse to the protoconid and has a tall and long posterior arm that fuses with the mesostylid, the latter of which bears a short accessory crest that terminates near the midpoint of the tooth. In DPC 16627, there is a short crest extending distally from the posterior position of the protoconid. The anterior arm of the entoconid is tall, but is interrupted by a small and shallow notch. The posterior arm of the protoconid varies from being a short crest to a small knob; it either protrudes from the protoconid or from the ectolophid, and is oriented toward the crest that extends labially from the mesostylid. In some individuals (Figures 12I and 13C), the metalophulid II unites with this crest, and forms a complete transverse crest that divides the anterior basin into two narrow valleys. The ectolophid is robust and runs distolingually from the protoconid, turning distally at its posterior part to merge with a long hypolophid. The latter is obliquely oriented, and together with the ectolophid forms a long sinuous course that divides the crown into two major basins. This diagonal crest is not seen in other Fayum rodents. The anterior arm of the hypoconid is interrupted by a shallow notch, but nevertheless connects the hypoconid to the junction of the hypolophid and the ectolophid. The posterior portion of the tooth is delimited by a posterolophid that curves distally and connects the hypoconid and the entoconid. In some cases, the lingual wall of the posterior basin bears a shallow notch. The crestiform hypoconid is oriented mesiolabially-distolingually and extends mesiolabially. Some individuals (e.g., DPC 16550) have a distinct ectostylid, but others lack this cusp altogether. There is no trace of an anterocingulid or a hypoconulid cusp, as occur in some other Fayum hystricognaths. The labial sinusid is narrow and deep.

The M_2_ occlusal surface is very similar to that of the first molar, differing in having relatively well-developed crests, a relatively wide trigonid, and in being relatively broad. In most individuals, M_2_ is larger than M_1_, but in DPC 20178, these loci are of about the same size. As on M_1_, the ectostylid is not found in all specimens, and ranges in size from being incipient (e.g., DPC 13823) to robust (e.g., DPC 16550). The posterior basin of the M_2_ sometimes bears either an accessory cusp or a small crest that runs from its lingual wall.

The M_3_ in most specimens is smaller than M_2_, but in DPC 20178 the teeth are roughly the same size. The M_3_ has a similar occlusal pattern to that of M_1–2_, but the middle crest is relatively straight, the posterior arm of the protoconid varies from being very short to absent, the lingual wall is relatively robust and continuous, and the accessory crest that runs from the lingual wall is relatively short or even cuspate. In some specimens (DPC 20513 and DPC 17632), the notch in the lingual wall of the posterior basin is wide and deep, whereas other specimens show little or no development of a notch. The anterior arm of the hypoconid is relatively weak or absent.

One upper tooth in the collection (Figure 9G, DPC 13196B) has an occlusal configuration similar to that of the M^1–2^ of *G. aslius*, but it is relatively long and large when compared with the M^1^ of that species. It is difficult to identify this tooth to locus with certainty, but the odd proportions, and the slightly different occlusal pattern, suggest that it might be a DP^4^ of *Gaudeamus aslius* rather than an aberrant upper molar. The anteroloph is slightly concave, suggesting that the mesial surface of the tooth might have accommodated an abutting DP^3^. The protoloph is relatively long and transversely oriented, and the middle crest is interrupted at its midpoint where the protoloph meets the anterior arm or the hypocone; this character is not seen on the M^1^s of *G. aslius* or *G. hylaeus*. The “mesoloph” is well-developed and runs lingually from the mesostyle to reach the most labial tip of the anterior arm of the hypocone. The metaloph is very short and directed toward the middle of the crown. The anterior arm of the metacone is tall and robust and fuses with the mesostyle, while the posterior arm of the paracone is absent, leaving a deep and wide notch in the labial wall that is not seen in *G. aslius*. A spur extends mesially from the middle of the posteroloph as in *G. aslius* and *G. hylaeus*.

### *Gaudeamus hylaeus*, sp. nov

urn:lsid:zoobank.org:act:1044F2AE-9EF6-43BE-AE8B-14D82E121B0B

**Figure 14:**
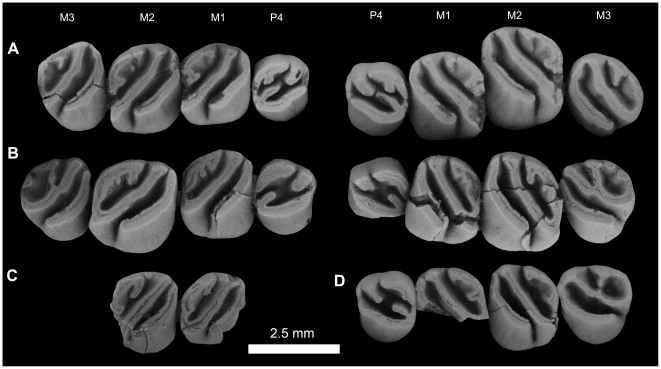
Upper dentitions of *Gaudeamus hylaeus*, sp. nov. **A**, DPC 15242, right P^4^-M^3^, left P^4^-M^3^; **B**, CGM 66007, right P^4^-M^3^, left M^1–3^; **C**, DPC 7772, right M^1–2^, broken; **D**, DPC 15147, left P^4^-M^3^ (M^1^ is broken). Apparent differences between two rows in the figure are due to the postmortem distortion and displacement of the crowns.

**Figure 15:**
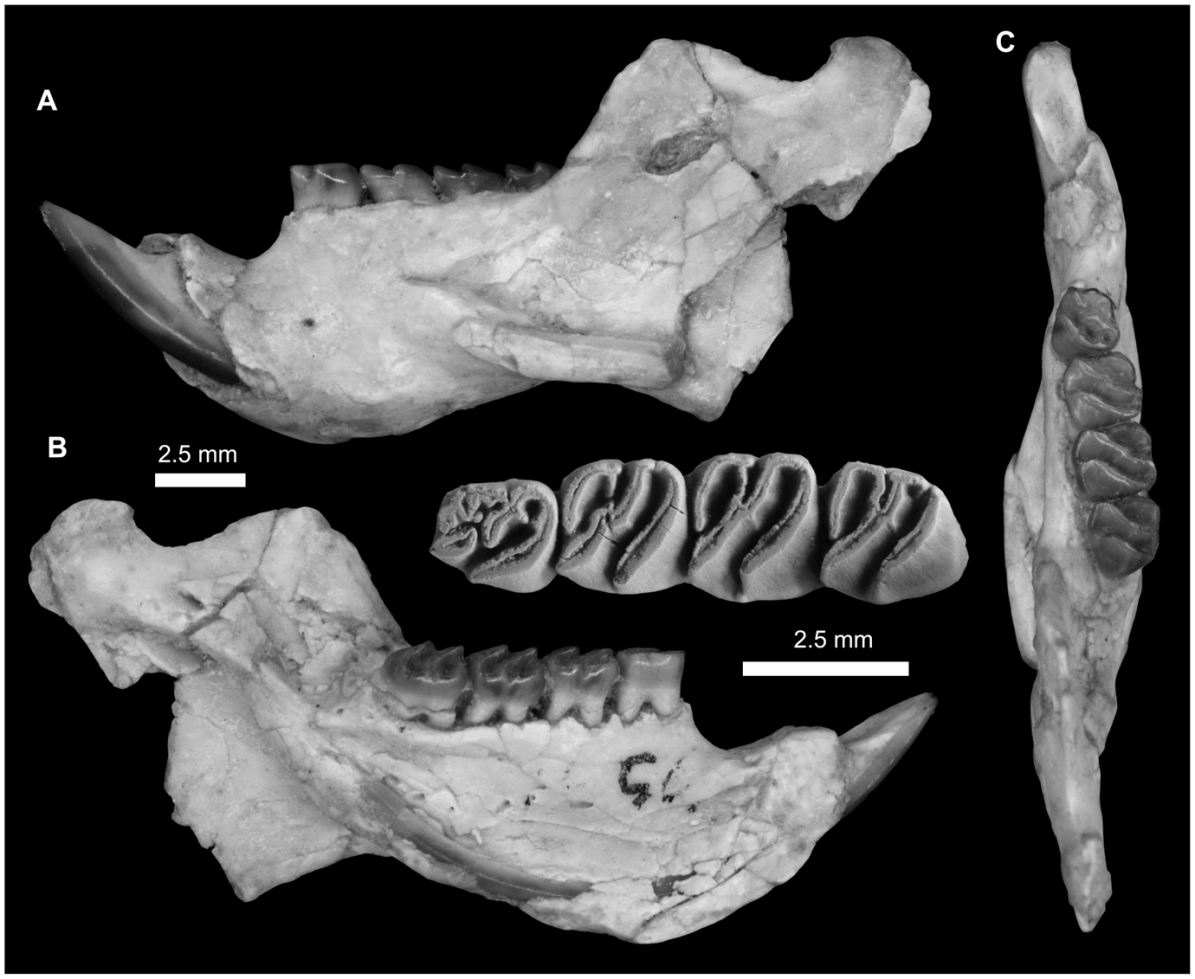
Mandibular fragments and lower dentition of *Gaudeamus hylaeus*, new species. **A–C,** DPC 9456, almost complete left mandible with P_4_-M_3_ and dislocated incisor. **A**, lateral view; **B**, medial view; **C,** occlusal view. Some of the mandible elements are displaced due to postdepositional distortion.

**Figure 16:**
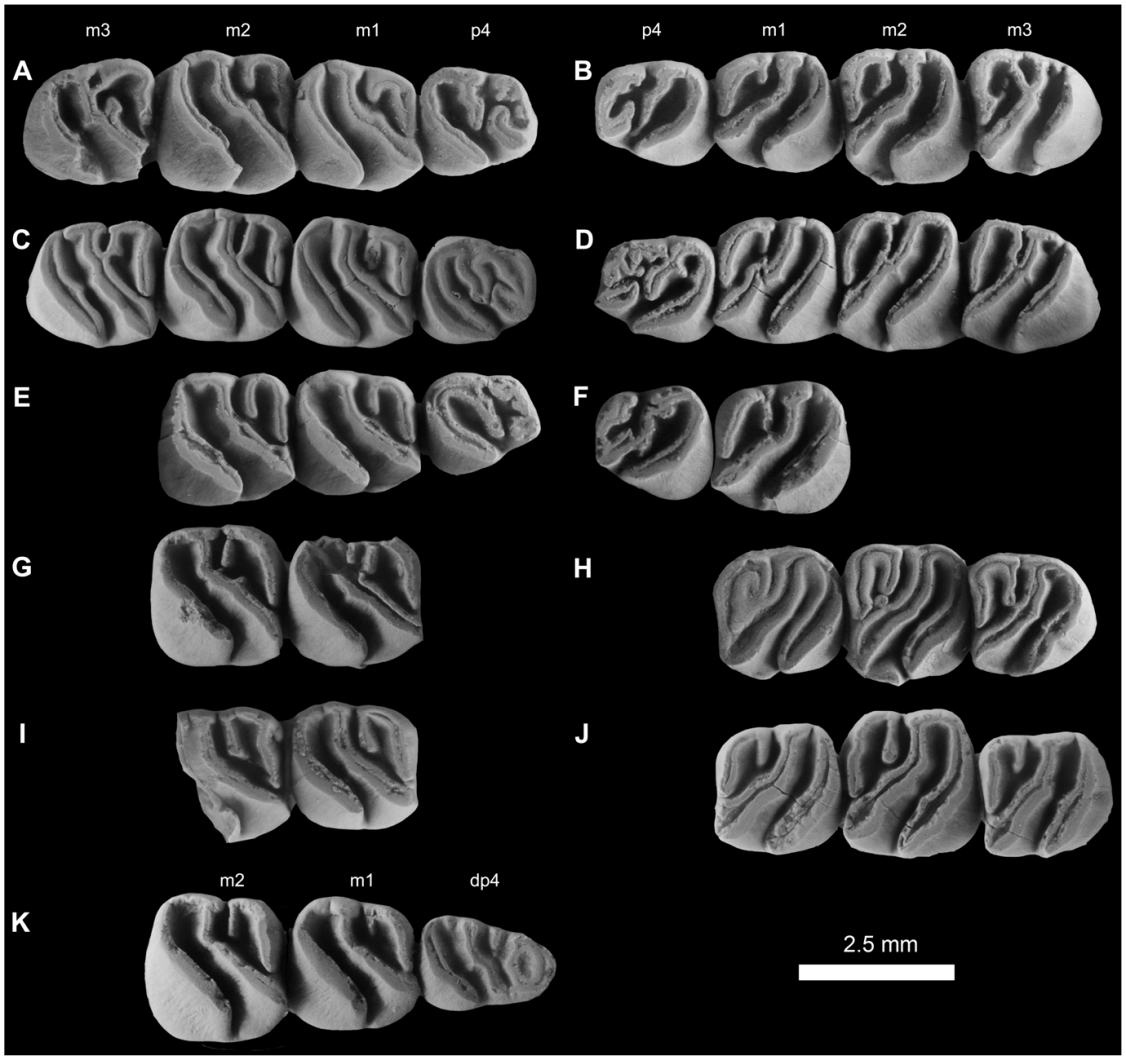
Lower dentitions of *Gaudeamus hylaeus*, new species. **A**, DPC 15406, right P_4_-M_3_; **B**, DPC 15181, left P_4_-M_3_; **C**, DPC D, right P_4_-M_3_; **D**, DPC 9456, left P_4_-M_3_; **E**, DPC 14428, right P_4_-M_2_; **F**, DPC 21315B, left P_4_-M_1_; **G**, DPC 16730, right M_1–2_; **H**, DPC 17872, left M_1–3_; **I**, DPC 17831, right M_1–2_; **J**, DPC 13161, right M_1–3_, reversed; **K**, DPC 16600, right dP_4_–M_2_.

Figures 14, 15, 16, 8B, Table 2

**Table 2:** Dental metrics for *Gaudeamus hylaeus*, sp. nov., and *Gaudeamus* aff. *hylaeus*, in millimeters.

*Gaudeamus hylaeus*											
Specimen	side	dP_4_		P_4_		M_1_		M_2_		M_3_	
DPC16600	right	2.25	1.53	-	-	2.15	2.20	2.18	2.38	-	-
DPC 9456	left	-	-	2.00	1.80	2.08	2.05	2.08	2.2	2.25	2.03
DPC 14428	right	-	-	1.95	1.75	2.13	2.10	2.25	2.23	-	-
DPC 15406	right	-	-	1.925	1.88	2.20	2.23	2.23	2.35	2.20	2.13
DPC 16730	right	-	-	-	-	2.18	-	2.05	2.13	-	-
DPC 17831	right	-	-	-	-	2.13	2.05	-	-	-	-
DPC 17872	left	-	-	-	-	2.01	1.90	2.05	2.23	2.05	1.88
DPC 21315	left	-	-	1.80	1.85	2.20	2.13	-	-	-	-
DPC 22693	right	-	-	1.88	1.85	2.01	2.08	2.00	2.15	2.05	2.05
DPC 13161	right	-	-	-	-	2.13	2.18	2.25	2.40	2.08	2.15

		dP^4^		P^4^		M^1^		M^2^		M^3^	
DPC 15242	left	-	-	1.58	2.00	1.95	2.33	2.13	2.60	2.00	2.20
	right	-	-	1.63	2.00	2.00	2.38	2.05	2.63	2.00	2.35
CGM 66006	left	-	-	1.75	-	2.00e	-	2.38e	-	2.13	2.13
	right	-	-	1.75	2.13	2.13	2.45	2.38	2.55	2.00	2.25
DPC 7772	right	-	-	-	-	-	2.18	-	2.43	-	-
DPC 15147	left	-	-	1.63	2.00	-	-	1.93	2.43	2.10	2.23

*Gaudeamus aff. hylaeus*											
		dP_4_		P_4_		M_1_		M_2_		M_3_	
DPC 17624	left	-	-	-	-	2.08	2.05	1.88	2.00	-	-
DPC 14487	right	-	-	-	-	2.08	2.08	1.95	2.20	-	-

Estimates are indicated by an “e”.

#### Etymology

From Holroyd [15], p. 133, “*hylaeus*, Latin, meaning of the wood or forest, savage, wild; in reference to the forested habitat that covered the Fayum region during the late Eocene and early Oligocene”.

#### Holotype

CGM 66007 (Figure 14B), a severely crushed (flattened) skull that preserves the whole upper dentition (right and left P^4^-M^3^ and upper incisors).

#### Type locality

Locality 41, lower sequence of the Jebel Qatrani Formation, Fayum Depression, Egypt.

#### Referred specimens

The hypodigm of *Gaudeamus hylaeus* includes two skull fragments in addition to the holotype, one maxillary fragment, isolated P^4^-M^3^ of the same individual, and 11 mandibular fragments: DPC 15242, skull fragment with right and left P^4^-M^3^ and a broken incisor; DPC 7772, a maxillary fragment with right M^1^-M^2^; DPC 15147, P^4^-M^3^isolated teeth but from the same individual; DPC 15406, a right mandibular fragment with P_4_-M_3_ and a complete incisor; DPC 15181, left mandibular fragment with P_4_-M_3_ and a broken incisor; DPC 22693, right mandibular fragment with P_4_-M_3_ and a broken incisor; DPC 9456, a left mandibular fragment with P_4_-M_3_, complete incisor, and condylar process; DPC 14428, a right mandibular fragment with P_4_-M_2_; DPC 21315B, left isolated P_4_-M_1_ for the same individual; DPC 16730, a right mandibular fragment with M_1–2_; DPC 17872, a left mandibular fragment with M_1–3_; DPC 17831, right mandibular fragment with M_1_, broken M_2_, and a complete incisor; DPC 13161, a right mandibular fragment with M_1–3_ and a broken incisor; and DPC 16600, a right mandibular fragment with dP_4_-M_2_.

#### Diagnosis

*Gaudeamus hylaeus* differs from *Gaudeamus aslius* in being relatively hypsodont, and in having relatively broad upper molars with a wide notch in the labial wall; a relatively large, crestiform, and distally placed protocone on P^4^ that forms most of the lingual border of the tooth; a relatively low and short P^4^ anteroloph that terminates mesial to the paracone, leaving a deep notch; a relatively oblique and short P^4^ protoloph that never crosses the midline of the tooth; a small P^4^ hypocone with a well-developed anterior arm that extends mesiolabially toward the protoloph; molar anterolophs and diagonal crests (i.e., protoloph + mure + anterior arm of the hypocone) that are relatively straight, and oblique with respect to the long axis of the tooth row; a deep valley separating the anteroloph and diagonal crest, with no development of a connection between the protocone and the protoloph (i.e., the taeniodont pattern); a relatively large M^1^; absence, or only slight development of, “mesolophs” on M^1–2^; upper molar metalophs that are either completely subsumed into posterolophs, or present as tiny spurs; a lingual sinus that is broadly open on M^3^; a dP_4_ that lacks a complete mesolophid, anterior arms of the hypoconid and entoconid, and posterior arm of the metaconid; a long and trenchant hypolophid on P_4_; lower molars that have relatively tall and more mesially inclined lophs, relatively straight diagonal crests, no anterior arm of the hypoconid, leading to a well developed taeniodont pattern, and relatively large M_1_ and M_3_; and mesiolingually tilted hypoconids.

Differs from *Gaudeamus aegyptius* in being large and more hypsodont, in having a longitudinal crest and a well-developed metalophulid I on P_4_; no 3-cusped crest or isolated metalophulid I on dP_4_; a straight diagonal crest and a relatively narrow notch on the anterior wall of the lower molars; a relatively long anterior arm of the hypocone and short and distolingual protoloph on P^4^; and a well-developed anterior arm of the metacone on upper molars.

*G. hylaeus* differs from *G. lavocati* in lacking the following combination of features: the crest that extends labially from the mesostylid is oriented mesially toward the metalophulid I on the lower molars; dP_4_ has a relatively complete mesolophid and anterior arm of hypoconid; M^1^ and M^2^ have long “mesolophs” that are directed distally; M^3^ is relatively broad; P^4^ lacks a complete endoloph, and bears a central crest “mesoloph”, a relatively weakly-developed and lingually placed metacone, a complete diagonal crest in the middle of the crown, and a relatively robust anteroloph.

### Description

Two partial crania of *Gaudeamus hylaeus* (CGM 66007 and DPC 15242) are severely crushed, and morphological features are either completely obscured or impossible to trace, but the tooth rows are well-preserved, despite some cracks.

#### Upper dentition

In general, the molars of *G. hylaeus* are relatively high-crowned. The upper teeth are wide and short, and M^1^ is relatively large when compared with that of *G. aslius*.

The P^4^ anteroloph is a low and short crest that runs labially from the protocone to terminate mesial to the paracone, where it is separated from that cusp by a deep notch. The protocone is the largest cusp on P^4^, is crestiform, placed approximately transverse to the metacone, mesiolabial-distolingual in orientation, and forms most of the convex lingual boarder of the tooth. The protoloph is very short and is distolingually oriented toward the anterior arm of the hypocone, from which it is consistently separated by a notch or a wide gap. The anterior and posterior basins are accordingly confluent and form a tilted “H”-shape in the center of the tooth. The posteroloph has a small notch along its lingual part.

The M^1^ is somewhat quadrate in outline. The protocone and anteroloph together form the mesial margin of the crown, which is relatively straight and more obliquely oriented than that of *G. aslius*. The anteroloph terminates as a parastyle on the mesiolabial margin. The protocone lacks any connection to the protoloph, leading to a well-developed taeniodont pattern. The protoloph, the mure, and the anterior arm of the hypocone together form a continuous diagonal crest which is straighter than that of *G. aslius*, and is separated from the parallel anteroloph by a deep valley which is open both lingually and labially. The metacone is relatively crestiform and is more lingually placed than that of *G. aslius*. The metaloph is completely subsumed into the posteroloph, but in CGM 66007 there is a short crest that protrudes from the posteroloph in this region. The configuration of the lingual wall between the paracone and the metacone is less complex than that of *G. aslius*; the anterior arm of the metacone is robust, as tall as the main crests, and connects to a large and well-developed mesostyle. A faint “mesoloph” variably descends lingually from the mesostyle, but never reaches the middle crest of the crown. The posterior basin is open labially due to the absence of the posterior arm of the paracone.

The M^2^ is the largest tooth of the upper dentition and has a similar occlusal morphology of that of M^1^, but has a relatively broad anterior portion and relatively narrow posterior portion. The metacone is in a slightly more lingual position than that on M^1^. The angle between the posteroloph and the diagonal crest at the position of the hypocone is very acute and the hypocone points distolingually.

The M^3^ of *G. hylaeus* has well-developed and obliquely oriented lophs, and is longer and relatively narrow in outline when compared with *G. aslius*. There is a large mesostyle on the labial border and a deep notch mesial to that cusp, and the “mesoloph” reaches the middle crest in most specimens. The lingual sinus is wide and deep and extends down to the base of the crown.

The mandible of *G. hylaeus* (Figure 15) is hystricognathous, robust, and dorsoventrally slender, with a relatively deep and long diastema when compared with those of *G. aegyptius* and *G. aslius*. The mental foramen is relatively small and placed under the P_4_ as in *G. aegyptius* and *G. aslius*. DPC 9456 preserves most of the mandible aside from the tips of the coronoid and angular processes, but the ventral masseteric ridge, angular process, and incisor were slightly distorted post-mortem.

#### Lower dentition

The dP_4_ of *G. hylaeus* is only known from DPC 16600 (Figure 16K). It is as large, and has the same basic occlusal pattern, as that of *G. aslius*, and is replaced by P_4_ early in life. The crown does not, however, have an incomplete mesolophid, and lacks the anterior arm of the hypoconid. Due to the absence of the latter crest, the labial sinusid is confluent with the posterior basin, forming an elongate and oblique basin that is relatively deep when compared with the anterior and middle basins. The diagonal crest is sinuous, and runs mesiolabially from the entoconid as a relatively short and oblique hypolophid that is fused with a relatively transversely oriented ectolophid. The mesostylid is situated midway between the metaconid and the entoconid and is a relatively well-developed cusp that is more isolated than that of *G. aslius*. There is no anterior arm of the entoconid or posterior arm of the metaconid, both of which are distinct in *G. aslius*.

The P_4_, is similar to that of *G. aslius* in size and morphology, but differs in having relatively tall lophs and cusps that are completely integrated into the three primary crests (metalophulid I, hypolophid, and posterolophid). The hypolophid is trenchant and runs mesiolabially from the entoconid, terminating between the protoconid and hypoconid and dividing the crown into anterior and posterior fossae. On DPC 21315B, the hypolophid connects to the posterior arm of protoconid. In some specimens the anterior basin is open lingually due to the presence of a deep notch on the lingual wall. The posterior basin is deeper than the anterior basin, and is generally open labially due to the absence of the anterior arm of the hypoconid, though in some specimens such as DPC 14428 and DPC 9456 the lingual wall is sealed off, apparently due to wear. In DPC 15406, the metalophulid I is interrupted labially by a shallow notch lingual to the protoconid, as in some *G. aslius* individuals. The posterolophid is completely fused to the entoconid, closing the posterior basin of the crown lingually. There is no ectolophid.

The M_1_ occlusal morphology is similar to that of *G. aslius*, but *G. hylaeus* has taller and more mesially inclined lophs, and a relatively short metalophulid I that terminates lingual to the protoconid, leading to a narrow crevice that leaves the anterior basin open mesially. In DPC 17831 and DPC 16600, the metalophulid I is weakly connected to the mesiolingually side of the protoloph as seen in *G. aslius*. The anterior arm of the entoconid is absent, leaving the anterior basin open lingually via a deeper notch than that seen in *G. aslius*. The metalophulid I, metaconid, posterior arm of the metaconid and the short crest that extends lingually from the mesostylid form a sharp hook-shaped wall on the anteriolingual portion of the crown. The primary diagonal crest is relatively straight when compared with that of *G. aslius*. In most specimens, the posterior arm of the protoconid is absent, but some individuals bear a faint spur that protrudes from the ectolophid in this area. The hypoconid is more crestiform than that of *G. aslius*, is oriented relatively mesiolabial-distolingual, tilts mesiolingually on the crown, and extends farther along the mesiolabial border than that of *G. aslius*. There is no anterior arm of the hypoconid, leaving a deep longitudinal basin that is continuous with, and deepens toward, the labial sinusid, forming a well-developed taeniodont pattern. DPC 17872 bears an ectostylid between the protoconid and the hypoconid. The posterolophid also has a relatively straight course when compared with *G. aslius*, running distolingually from the hypoconid and then curving mesially along its lingual border to either fuse with the entoconid or terminate just distal to that cusp.

The M_2_ occlusal surface is nearly identical to that of M_1_, differing only in being relatively large and broad, having a relatively wide trigonid, and more commonly bearing an ectostylid cusp. The lingual wall of the posterior basin is always closed by a high crest that is sometimes interrupted by a very shallow notch.

The M_3_ varies in its size relative to M_2_, and has a similar overall occlusal pattern to that tooth, but its posterior portion is relatively narrow. The hypolophid is relatively short, and the ectolophid relatively long, when compared with M_1–2_. In most specimens the crest that runs labially from the mesostylid turns distally to contact with the most lingual part of hypolophid.

### *Gaudeamus* aff. *aslius*

Figure 17; Table 1

**Figure 17:**
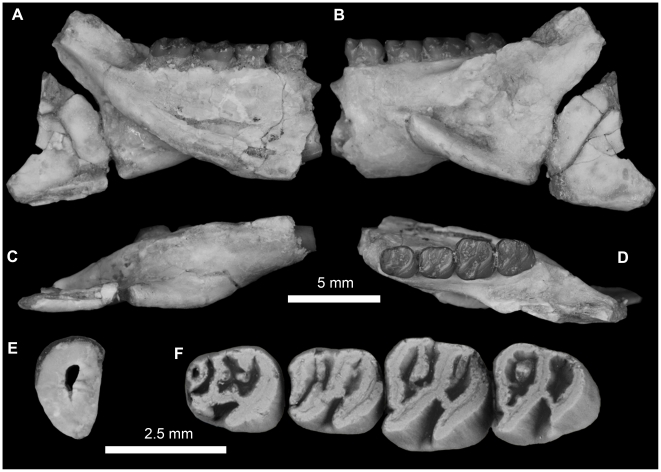
Mandibular fragment and lower dentition of *Gaudeamus* aff. *aslius*. **A–F,** DPC 12990, fragment of left mandible with P_4_-M_3_ and broken incisor. **A**, medial view; **B**, lateral view; **C** ventral view; **D,** occlusal view; **E**, cross section of incisor; **F**, close up in occlusal of P_4_-M_3_.

**Figure 18:**
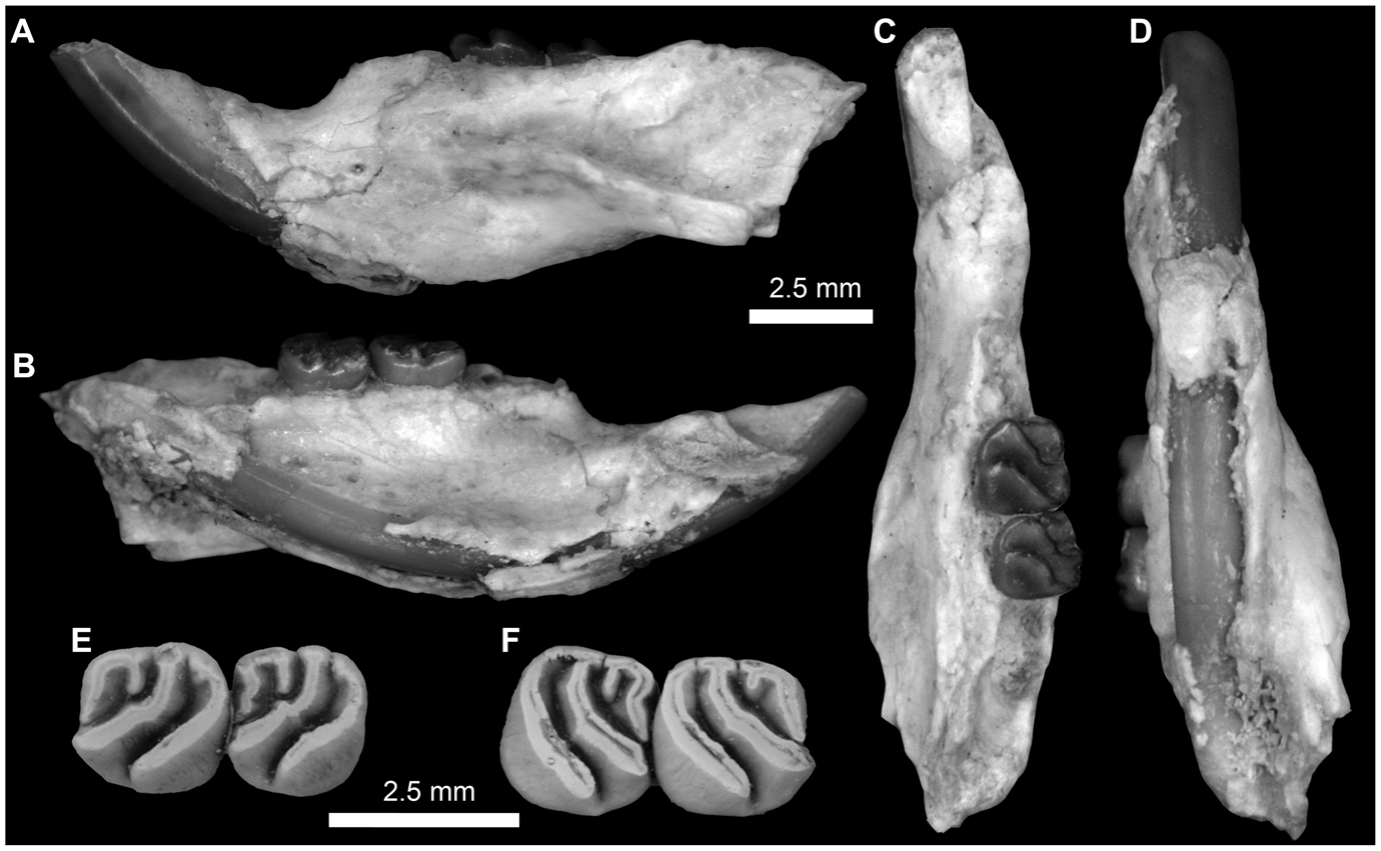
Plot of lower first and second molars areas in *Gaudeamus* spp., with 95% confidence intervals, showing unique proportions of *Gaudeamus* aff. *aslius*.

A single lower jaw fragment with P_4_-M_3_ and an almost complete angular process (DPC 12990) exhibits occlusal morphology that is very similar to that of *G. aslius*, but differs from all specimens in that species' hypodigm in having a very small M_1_ and a relatively large P_4_. The size difference between the area of M_1_ and that of M_2_ falls well outside the 95% confidence intervals for the specimens that we recognize as *G. aslius* (Figure 18). Because a similar discrepancy in the size of M_1_ and M_2_ also occurs in *Waslamys attiai*, one of the oldest and most primitive Fayum hystricognaths [16], it is conceivable that this molar size discrepancy is a primitive feature retained in an additional new species of *Gaudeamus*, and is not simply due to intraspecific variation. We conservatively refer to this specimen as *Gaudeamus* aff. *aslius*. The teeth of this specimen are low-crowned, have relatively transversely oriented hypolophids, complete ectolophids in lower molars, wide P_4_ sinusids, and a well-developed anterior arm of the hypoconid on M_2_ and M_3_. The protoconid and hypoconid on P_4_ are connected by a complete ectolophid, and a complete posterior arm of the protoconid reaches the lingual wall and fuses with the mesostylid. The P_4_ further differs from those of *G. aslius* in having a relatively long hypolophid, a wider sinusid, and an incipient anterocingulid. An accessory crest runs from the metaconid toward the anterior margin of the tooth and fuses with the accessory cusp on the middle of the metalophulid I, forming a small fovea at the mesiolingual corner of the crown. At the junction of the anterior arm of the hypoconid and the ectolophid is a small projection protruding lingually toward the entoconid. This lophule could be a remnant of the labial part of the hypolophid. The mandibular morphology and occlusal pattern on M_1–3_ is similar to that of *G. aslius*.

### *Gaudeamus* aff. *hylaeus*

Figure 19; Table 2

**Figure 19:**
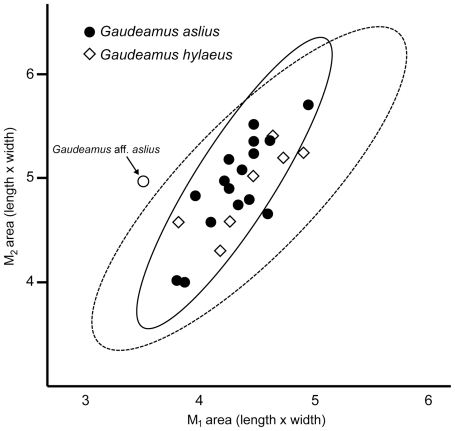
Mandibular fragment and lower dentition of *Gaudeamus* aff. *hylaeus*. A–E, DPC 17624, fragment of left mandible with broken incisor. A, lateral view; B, medial view; C occlusal view; D, ventral view; E, close up in occlusal view of M_1–2_. F, close up in occlusal view of right M_1–2_ of DPC 14487.

Two specimens are assigned to this taxon. DPC 17624 is a mandibular fragment with a broken incisor. The dP_4_, M_3_, and the posterior part of the mandible (coronoid, angular, and condylar processes) is missing. DPC 17624 represents a relatively young individual because it has a relatively slender mandible with a shallow corpus, a dP_4_ alveolus, a short diastema, a thin incisor, and the molars are relatively unworn. The mandible shows slight post-mortem distortion. DPC 17624 shows some occlusal morphological resemblances to *G. hylaeus* in having a relatively high crown, a short and labially interrupted metalophulid I, and a relatively straight diagonal crest, and in lacking an anterior arm of the hypoconid and an anterior arm of the entoconid. The specimen differs from that of *Gaudeamus hylaeus* in being smaller, in having an M_2_ that is smaller than M_1_, and in having a relatively lingually positioned protoconid. Furthermore, M_2_ is relatively narrow, its metalophulid I is curved distally and the entoconid is slightly lingual in position relative to the metaconid.

DPC 14487 is a badly broken mandible with a complete incisor and M_1–2_. It has the same occlusal configuration as that of DPC 17624. The teeth and the incisor are slightly larger than those of DPC 17624, and the M_1_ is the same size as the M_2_. In addition, the lingual wall is somewhat sealed.

### Phylogenetic analysis

**Figure 20:**
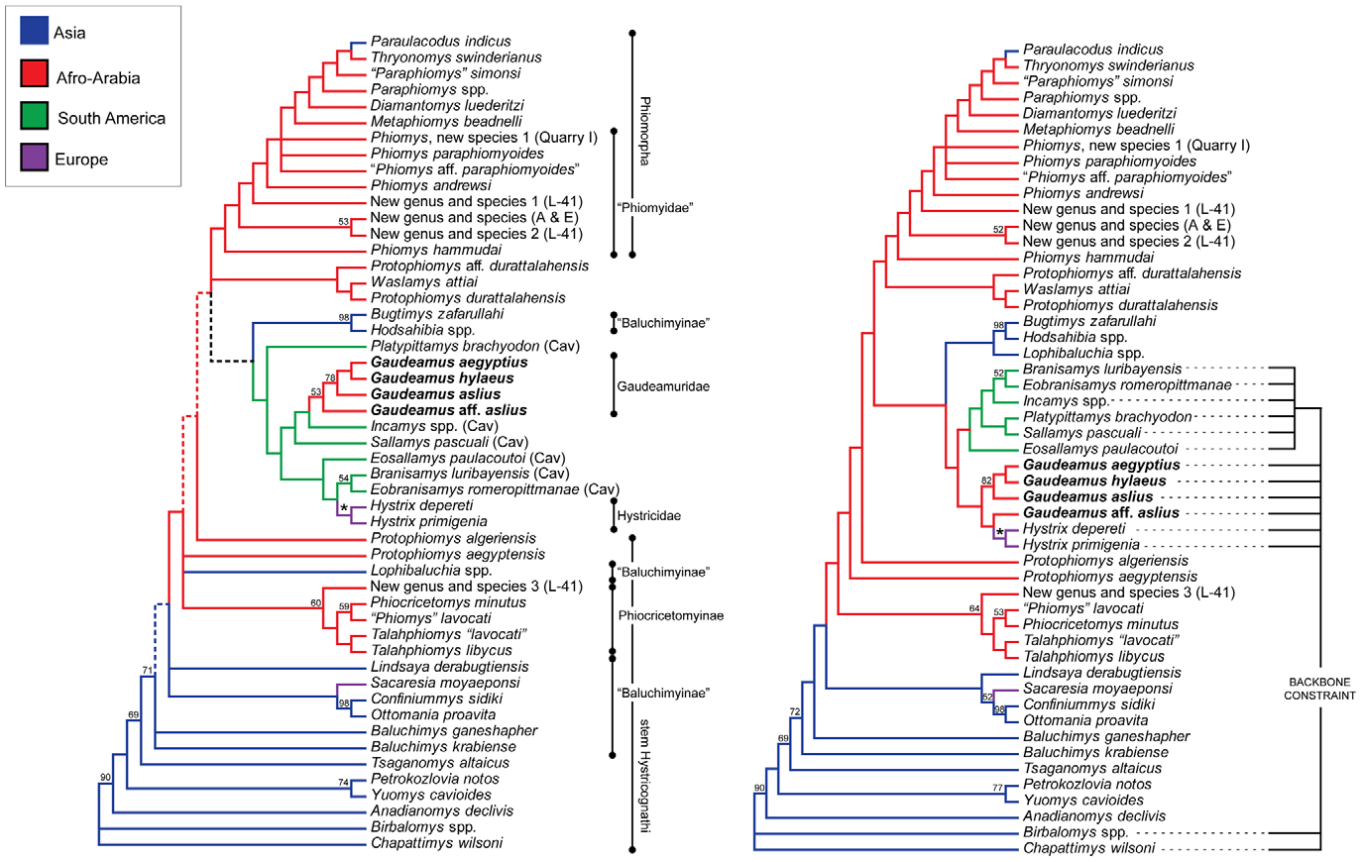
Phylogenetic analysis of living and extinct hystricognathous rodents, based on 118 morphological characters, largely from the dentition. A) Adams consensus tree derived from unconstrained parsimony analysis with some multistate characters ordered and scaled; branches with dotted lines break down in the strict consensus tree. Consensus trees are based on 31 equally parsimonious trees of length 458.12502; consistency index excluding uninformative characters = 0.2959; retention index = 0.6046; rescaled consistency index = 0.1854. B) Strict consensus tree based on 31 equally parsimonious tree recovered from parsimony analysis with caviomorph monophyly constrained (backbone constraint shown to the right of the tree); tree length = 459.87502; consistency index excluding uninformative characters = 0.2948; retention index = 0.6024; rescaled consistency index = 0.1841. Taxa labelled as “Cav” in (A) are universally considered to be fossil members of Caviomorpha. On both trees, numbers above branches are bootstrap support values based on 1000 replicates; “*” indicates bootstrap support of 100. Biogeographic histories are based on parsimony optimizations of an unordered biogeographic character with four states (Asia, Afro-Arabia, South America, Europe) onto the Adams consensus tree (a) and strict consensus (b).

**Figure 21:**
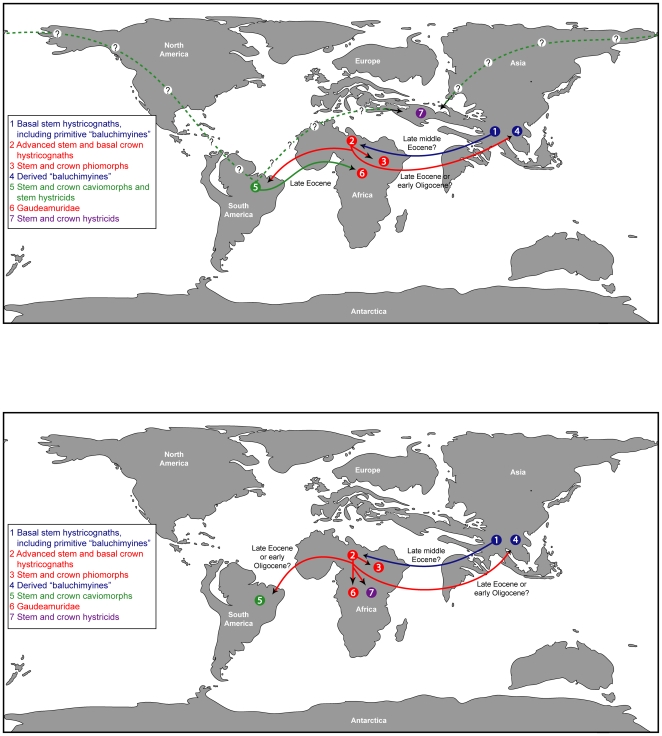
Dispersal routes of early hystricognaths implied by the results of phylogenetic analyses presented in **Figure 20**. A) Dispersal routes implied by the unconstrained analysis of morphological data (see Fig. 20A), requiring a back-migration from South America to Afro-Arabia to account for the presence of *Gaudeamus* in the late Eocene of Egypt. B) Simpler biogeographic scenario implied by the phylogenetic analysis of morphological characters with caviomorph monophyly constrained (see Fig. 20B). Eocene paleogeographic map is modified from http://jan.ucc.nau.edu/~rcb7/mollglobe.html.

A parsimony analysis of morphological features was undertaken in order to test existing hypotheses of *Gaudeamus*' placement within Hystricognathi – i.e., close affinities with *Thryonomys* [14], [24], Caviomorpha, or Hystricidae. Unconstrained parsimony analysis with some multistate characters ordered and scaled placed *Gaudeamus* as the sister group of the late Oligocene South American caviomorph *Incamys*, followed by the South American caviomorph *Sallamys* (Fig 20a). This arrangement is slightly different from that which was recovered by Sallam et al. 's unconstrained parsimony analysis based on a more limited taxon sample, which placed *Gaudeamus* as a sister group of a (*Eoincamys*, (*Branisamys*, *Eobranisamys*)) clade. The consensus tree nests *Gaudeamus* and Hystricidae firmly *within* the South American hystricognath radiation, rendering Caviomorpha paraphyletic with respect to Hystricidae, and would require at least two late Eocene (or earlier) dispersals across the Atlantic Ocean (one from Africa to South America to account for the presence of caviomorphs in South America, and one from South America back to Africa to account for the presence of *Gaudeamus* on the latter continent), and one later dispersal out of South America (either overland or overwater) to account for the presence of hystricids in the Old World (Fig. 21a). These phylogenetic patterns could also be explained by three long-distance overland dispersals through northern continents (one to explain the origin of Caviomorpha, one to explain a back-dispersal of *Gaudeamus* to Africa, and one to explain the Old World distribution of Hystricidae), but there is currently no evidence for any such dispersals in the fossil records of Europe, Asia, or North America. Unlike the unconstrained analysis of Sallam et al. [16], in the current analysis *Thryonomys* was placed in a nested position within Phiomorpha as the sister group of *Paraulacodus*, as previously suggested by Flynn and Winkler [34]. Primitive African and Asian taxa such as *Protophiomys*, *Waslamys*, phiocricetomyines, and “baluchimyines” were generally placed in a more basal position in the tree as stem hystricognaths, but in the Adams consensus tree two derived baluchimyines (*Bugtimys* and *Hodsahibia*) were placed within the hystricognath crown clade as the sister group of the Caviomorpha-Hystricidae-*Gaudeamus* clade to the exclusion of phiomorphs.

Many students of hystricognath systematics would likely argue that the unconstrained parsimony analysis of morphological data recovered a topology that incorrectly challenges several well-founded hypotheses of relationships within Hystricognathi, such as a monophyletic Caviomorpha that includes all of the early South American taxa included here [29], [35]. Indeed, molecular data strongly suggest that Hystricidae is not nested within Caviomorpha but rather is a sister group of a Caviomorpha-Phiomorpha clade [36]. There are compelling reasons to question the likelihood of the most parsimonious unconstrained topology; while this topology minimizes *ad hoc* hypotheses of morphological homoplasy, it is important to consider that 1) it does so almost entirely in only one very rapidly evolving morphological region (the dentition), and that 2) relative to the well-founded hypothesis of caviomorph monophyly, the most parsimonious unconstrained topology presented here significantly increases the number of non-morphological *ad hoc* hypotheses that are required to account for overwater dispersals and/or non-preservation in the fossil record. With regard to the trans-Atlantic dispersal *from* South America *to* Afro-Arabia that is implied by the position of *Gaudeamus* within Caviomorpha, it is also important to consider that no other examples of such a dispersal pattern are indicated in the Eocene or Oligocene fossil records of either Afro-Arabia or South America, whereas westward dispersal *from* Afro-Arabia *to* South America is now generally considered to be the likely mechanism for transport of both ancestral caviomorphs and ancestral platyrrhine anthropoid primates to the New World.

In light of these considerations, we also ran a parsimony analysis with the monophyly of the early South American hystricognaths (i.e., presumed basal caviomorphs) constrained (Fig. 20b); the purpose of this secondary analysis was to control for the possibility that homoplasy in dental features in highly derived taxa such as *Hystrix* led to phylogenetic inaccuracy in the unconstrained analyses, and that this inaccuracy in turn might have affected the placement of *Gaudeamus*. Importantly, when monophyly of the presumed fossil caviomorphs was constrained, *Gaudeamus* was not placed as a stem caviomorph, but rather was placed as the sister taxon of *Hystrix*; *Gaudeamus* aff. *aslius* was placed as the sister taxon of *Hystrix* to the exclusion of other *Gaudeamus* species (Fig. 20b). This result indicates that *Gaudeamus*' placement in Caviomorpha was driven in part by its attraction to *Incamys*, a very derived genus that is not representative of the caviomorph morphotype. Other important rearrangements relative to the unconstrained tree included placement of the derived Asian baluchimyines *Bugtimys*, *Hodsahibia*, and *Lophibaluchia* in a clade which formed the sister group of a Caviomorpha-Hystricidae clade (implying a trans-Tethyan dispersal from Afro-Arabia to Asia), and the shift of *Waslamys* and “*Protophiomys*” *durattalahensis* to the base of the phiomorph clade (Fig. 21b). Otherwise, relationships within Phiomorpha corresponded precisely with those in the unconstrained tree. Comparing the results of the unconstrained analysis and the constrained analysis using a Templeton tests in PAUP* 4.0b10 reveals that the constrained tree is minimally longer and could not be rejected (unconstrained tree (tree length (TL) = 457.12502) to constrained tree (TL = 458.87502), *P* = 0.6367).

## Discussion

*Gaudeamus* has long been — and, despite the recovery of new material, continues to be — the most enigmatic of the Fayum Rodentia. The genus has most commonly been interpreted as the ancestral stock for the extant African cane rat *Thryonomys* [14], [24], but *Gaudeamus* also shares dental features with some early South American caviomorphs, notably the very derived forms *Incamys* and *Eoincamys* [21], [37]. More recently, the parsimony and chronobiogeographic analyses of Sallam et al. [16] placed *Gaudeamus* as either a sister group of hystricids, or nested within Caviomorpha, but never with the phiomorph *Thryonomys*; the parsimony analyses presented by Coster et al. [21] similarly placed *Gaudeamus* within Caviomorpha. In light of the new material described here, and our phylogenetic results, we discuss each of these hypotheses in turn below.

### The *Thryonomys* hypothesis

As originally noted by Wood [14], *Gaudeamus* does exhibit a number of similarities to *Thryonomys* in the occlusal morphology of its upper and lower teeth, and the cladistic analyses of Antoñanzas et al. [24] actually found *Thryonomys gregorianus* to be more closely related to *Gaudeamus* than to its congener *Thryonomys swinderianus*. However, numerous additional lines of evidence run contrary to the hypothesis of a close link between *Gaudeamus* and *Thryonomys* to the exclusion of all other Eocene-to-Recent rodents.

An important non-morphological consideration is *Gaudeamus*' age. The genus first appears at the ∼34 Ma Quarry L-41, but the molecular dating analyses of Sallam et al. [16], which otherwise exhibit good concordance with the hystricognath fossil record, found the split between *Thryonomys* and *Petromus* to have occurred around 18 Ma – that is, well into the Miocene. Based on these results, *Gaudeamus* is about two times older than would be expected for an exclusive sister taxon of *Thryonomys*. Furthermore, an exclusive relationship of *Thryonomys* and *Gaudeamus* to the exclusion of Miocene taxa such as *Apodecter*, *Paraulacodus*, *Paraphiomys* would require a 27-million-year-old ghost lineage for *Thyronomys* which first appears in the fossil record at ∼6 Ma in east Africa [38], through the very well-sampled early Oligocene and Miocene strata of northern and eastern Africa, while the lineages leading to *Apodecter*, *Paraulacodus*, and *Paraphiomys* would themselves each have to trace back beyond the late Eocene levels where the oldest *Gaudeamus* species have been recovered, and where such fossils have never been found.

One of the most important morphological differences between the two genera is the retention of dP_4_ and dP^4^ throughout life in *Thryonomys* [32], whereas dP_4_ and dP^4^ are clearly lost early in life in *Gaudeamus*. The late retention of dP_4_ and dP^4^ is seen in derived early Oligocene phiomorphs such as *Metaphiomys*, and is a consistent feature of fossil phiomorphs through the Oligocene and Neogene [14], [30]. This feature appears to be a key synapomorphy of derived Phiomorpha that is lacking *Gaudeamus*. Though the molars of *Gaudeamus* and *Thryonomys* undoubtedly show great overall similarity, *Thryonomys* most obviously differs from *Gaudeamus* in having cusps that are completely integrated into tall lophs. Furthermore, *Thryonomys* retains a longer and more transversely oriented protoloph that is connected to the protocone; together with the well-developed anterior arm of the hypocone, this crest forms an L-shaped middle crest rather than a diagonal crest as in younger *Gaudeamus*. The protocone of *Thryonomys* is also relatively lingual in position with respect to the hypocone, and the “mesoloph” and mesostyle of the upper molars are absent; the lower molars of *Thryonomys* lack the mesostylid and its accessory crest, the relatively well-developed lingual wall seen in *Gaudeamus*, and have well-developed anterior arms of the hypoconid. In addition, the lower molars of *Thryonomys* lack the metalophulid II that is well-developed in *Gaudeamus aslius*. The dP_4_ of *Thryonomys* [14] also lacks a well-developed metalophulid II whereas this crest is present in *Gaudeamus*. Aside from obvious differences in body size, *Thryonomys* also differs from *Gaudeamus* in having a series of potentially and unambiguously derived features, such as the lack of a well-developed postorbital process, a relatively high coronoid process, a well-developed ventral ridge of the masseteric fossa, upper incisors with grooved enamel surfaces, and lower incisors that are covered with a relatively a thin enamel layer. In our opinion, *Thryonomys* shows greater similarity to younger genera such as *Paraphiomys* and *Paraulacodus* (with which it forms a clade in our phylogenetic analyses) than to the much more ancient *Gaudeamus*, particularly given that *Paraulacodus* shares grooved incisors with *Thryonomys* [39], and such a relationship does not require the 27-million-year-old ghost lineage implied by an exclusive relationship of *Gaudeamus* to *Thryonomys* [14], [30].

### The caviomorph hypothesis

*Gaudeamus* bears clear similarities in dental morphology to some early South American caviomorphs, though we suspect that at least some of these similarities, particularly those shared with derived taxa such as *Eoincamys* and *Incamys*, evolved in parallel on the two continents. *Gaudeamus aslius* exhibits a combination of dental similarities to *Eosallamys paulacoutoi*, one of the earliest known fossil caviomorphs from the latest Eocene or Oligocene of Peru [37], and *Sallamys pascuali*, from the Oligocene of Bolivia, in having a similar overall configuration of crests on the upper and lower teeth — specifically in having a sinuous course of the diagonal crest on the upper molars (because of the more lingual orientation of the protoloph portion), a well-developed anterior arm of the hypoconid and metalophulid I on the lower molars, and a connection between the protoloph and protocone. Interestingly, the dP_4_ of *Eosallamys* (in particular specimens LACM 143420 and 143450) has the same pentalophodont occlusal pattern as that of *G. aslius*, including a well-developed mesolophid and mesostylid, but the dP_4_ of *Sallamys* differs from that of *G. aslius* in having an ectolophid that connects to metalophulid II rather than to the protoconid, and a crest that runs labially from the midpoint of the ectolophid. Lower and upper permanent premolars of *Eosallamys* show major differences from those of *G. aslius* in having a tetralophodont pattern and a complete protoloph and metaloph on P^4^, and a metalophulid II and anterior arm of the hypoconid and hypolophid on P_4_. Furthermore, the upper molars of *G. aslius* differ in having a weakly-developed connection between the protoloph and protocone, a short “mesoloph” that is usually oriented posteriorly, and a relatively small M^3^. The differences between *G. aslius* and *Sallamys* are basically the same as noted above for *Eosallamys*, except that *Sallamys* is relatively large and has relatively high-crowned teeth.

*Gaudeamus hylaeus* is very similar to latest Eocene or Oligocene *Eoincamys pascuali*, from the Santa Rosa locality in Peru [37], and late Oligocene *Incamys bolivianus* from Bolivia [29] in having a mesiolabial-distolingual orientation of lophs and a straight course of the diagonal crest on the upper molars; a complete endoloph on P^4^; in lacking a protoloph-protocone connection, leading to a well-developed transverse sinus between the anteroloph and the diagonal crest; and in having a metaloph and complete endoloph on the upper molars. *G. hylaeus* differs in lacking the anterior arm of hypoconid on the lower molars, leading to a transverse sulcus between the middle crest and the posterolophid; lacking the anterior arm of the entoconid; and having a well-developed connection of the metalophulid with the protoconid, forming an inverted hook-shape in the anterior part of the lower dentitions. *G. hylaeus* shows further differences from *Eoincamys pascuali* in having relatively high-crowned cheek teeth and well-developed unilaterally hypsodont upper dentitions; relatively crestiform cusps on the lower and upper molars; a short “mesoloph” that is oriented lingually from the mesostyle; a relatively weakly-developed and incomplete middle crest on P^4^; and a hypolophid connecting to the ectolophid in P_4_. The *G. hylaeus* dP_4_ is radically different from that of *Eoincamys pascuali* LACM 143335 [37] in being long, having a pentalophodont occlusal surface, and lacking a well-developed metalophulid I and mesostylid. *Incamys* has more hypsodont teeth when compared with those of *G. hylaeus*, and *Incamys* lacks a P^4^ endoloph and has an isolated mesostylid on P_4_ that are not present in *G. hylaeus*.

The primary problem with the caviomorph hypothesis is that the most parsimonious placement of *Gaudeamus* is deep *within* Caviomorpha, hence requiring a trans-Atlantic dispersal back to Africa, and notably during a time period (late Eocene) when there are, in fact, potentially no caviomorphs in South America (the Santa Rosa locality's purported late Eocene age being based on “stage of evolution” biostratigraphy). The oldest well-dated caviomorphs are about 31.5 Ma and hence early Oligocene in age [40], from a horizon that is younger than the latest occurrence of *Gaudeamus* in the Fayum succession (at about 33 Ma); the recently described *Gaudeamus lavocati* from Zallah, Libya, might be as young as the oldest well-dated caviomorphs, but is also the most derived *Gaudeamus* species known. As such, the placement of *Gaudeamus* deep within Caviomorpha also requires numerous ghost lineages for caviomorphs in South America. Finally, the most parsimonious placement of *Gaudeamus* outside of a monophyletic Caviomorpha is not on the caviomorph stem lineage but as sister to Hystricidae; constraining *Gaudeamus* to be the sister group of a monophyletic Caviomorpha requires an additional ∼2.5 steps relative to the analysis that only constrains caviomorph monophyly, but the tree was not rejected by a Templeton test (unconstrained tree (tree length (TL) = 457.12502) to constrained tree (TL = 461.29168), *P* = 0.5178).

### The hystricid hypothesis

A close relationship of *Gaudeamus* to hystricids is a novel hypothesis that was first recovered in the chronobiogeographic analysis presented by Sallam et al. [16], but not discussed in depth by the authors of that study. On the strict consensus tree derived from our analysis with caviomorph monophyly constrained, a total of 11 character state changes are optimized as synapomorphies of the *Gaudeamus*-hystricid clade, eight of which optimize unambiguously (Table 3), although most of these are changes from polymorphic to “fixed” states. Regardless, the hypothesis is not without character support.

**Table 3:** Morphological character support for the *Gaudeamus-Hystrix* clade depicted in Figure 20b.

Character no.	Character state change
character 7	interprismatic matrix (IPM) of incisor enamel thin ⇒ IPM thick (ambiguous optimization)
character 14	dP_4_ posterior arm of the metaconid polymorphically weak and low/well-developed and high ⇒ well-developed and high (ambiguous optimization)
character 22	dP_4_ anterior arm of hypoconid strong ⇒ polymorphically weak/absent (unambiguous)
character 32	P_4_ metalophulid I polymorphically incomplete, unconnected to metaconid/complete ⇒ complete
character 43	M_1_ mesostylid polymorphically weak/strong ⇒ strong (unambiguous)
character 50	M_1–2_ direction of the posterior arm of the protoconid directed toward metaconid ⇒ oblique, extending backward (unambiguous)
character 53	M_1–2_ metalophulid II polymorphically well-developed/weak ⇒ weak (unambiguous)
character 55	M_1–2_ anterior arm of hypoconid strong ⇒ polymorphically connects weakly to mesoconid/strong (unambiguous)
character 56	M_1–2_ entoconid anterior to hypoconid ⇒ entoconid and hypoconid opposed (unambiguous)
character 83	P^4^ hypocone polymorphically weak/strong ⇒ strong (unambiguous)
character 104	M^3^ metaloph polymorphically turned posteriorly to join posteroloph/turned posteriorly to join posteroloph ⇒ turned posteriorly to join posteroloph (ambiguous)

One attractive aspect of the *Gaudeamus*-hystricid link is that such a relationship would finally help to fill in the extensive ghost lineage for hystricids, which otherwise would be ∼28 million years long, given the molecular divergence estimate of ∼39 Ma for the split of Hystricidae from Caviomorpha-Phiomorpha provided by Sallam et al. [16]. The first record of Hystricidae in the fossil record is from ∼11 Ma deposits in Egypt [41]. In our constrained analysis, *Gaudeamus* species aside from *Gaudeamus* aff. *aslius* form a clade to the exclusion of *Hystrix*, and hence the more specialized dental features typically associated with *Gaudeamus* would be autapomorphies of that side branch, and not ancestral for Hystricidae, but the direct connection of *Gaudeamus* aff. *aslius* and *Hystrix* could indicate that the morphology of the former species might approximate the hystricid morphotype. Clearly much more complete material from the cranium and postcranium will be needed to provide a more compelling test this interesting hypothesis, but at present we consider a *Gaudeamus*-hystricid link to be more likely, given biogeographic considerations, than a nested placement of *Gaudeamus* or Hystricidae deep *within* the caviomorph clade, as is suggested by the unconstrained topology.

### Origin of Gaudeamuridae

It is very likely that the highly derived occlusal pattern of *G. aegyptius*, *G. hylaeus*, and *G. lavocati* is derived from a more distant common ancestor that resembled *Gaudeamus aslius*. The former taxa all have derived features, such as increased hypsodonty; a well-developed entoconid-protoconid crest on the lower molars; complete loss of the anterior arm of the hypoconid and the connection between the protocone and the protoloph; and a relatively straight crest connecting the paracone and hypocone crest on the upper molars. The identification of at least two, but possibly as many as four, species of *Gaudeamus* of roughly the same size at the ∼34 Ma Quarry L-41, and the lack of *Gaudeamus* at the ∼37 Ma Locality BQ-2 and the presumably mid-to-late Priabonian Idam Unit at Dor el-Talah, Libya [22], which we suspect is no more than ∼2 Ma older than Quarry L-41, suggests a mid-to-late Priabonian (late Eocene) origin, and subsequent rapid radiation, of gaudeamurids. The genus is very common at Quarry L-41, but disappears locally from the fossil record a few million years into the early Oligocene. A richer understanding of *Gaudeamus*' appearance, the adaptive basis for the rapid evolution of gaudeamurid hypsodonty, and the climatic, environmental, or competitive basis for the group's subsequent demise – apparently occurring over a geologically short period of time from ∼36 to ∼32 Ma — will require a high-resolution record that is not yet available in the African fossil record.

## Supporting Information

Appendix S1 Characters and character states employed in phylogenetic analyses.
(DOCX)

Appendix S2 Character-taxon matrix employed in phylogenetic analyses.
(DOCX)
