# Supplementary material for: Craniodental Morphology and Systematics of a New Family of Hystricognathous Rodents (Gaudeamuridae) from the Late Eocene and Early Oligocene of Egypt
Source: PLoS One. 2011 Feb 22;6(2):e16525. doi: 10.1371/journal.pone.0016525 (PMC3043065; doi:10.1371/journal.pone.0016525)
Supplement: Appendix S1 — Characters and character states employed in phylogenetic analyses. (DOCX) [file pone.0016525.s001.docx]

**Appendix S1**

Modified character set of Marivaux et al. (2004) and Sallam et al. (2009) employed in phylogenetic analyses. States such as “0/2” are polymorphisms (i.e., taxa scored as such exhibit both state 0 and state 2).

1. infraorbital foramen

0) protrogomorphous; 1) hystricomorphous

2. mandible

0) sciurognathous; 1) hystricognathous

3. I2/i2) enamel disposition

0) surrounds incisor; 1) does not totally surround incisor; 2) limited to buccal surface

4. Hunter-Schreger bands

0) absent; 1) present

5. prisms per Hunter-Schreger band

0) 1-3; 1) 0/2; 2) 4-6; 3) 2/4; 4) 7 or more

6. IPM crystallites arrangement in PI

0) sheath surrounding prisms; 1) 0/2; 2) parallel; 3) 2/4; 4) acute angle, anastomosing; 5) 4/6; 6) perpendicular

7. IPM thickness

0) thick; 1) thin

8. percentage of PE thickness

0) PE<=PI; 1) PE reduced (<25%); 2) PE lacking

9. band inclination relative to EDJ

0) 0 degrees; 1) >10 degrees; 2) 10-19 degrees; 3) 20-29 degrees; 4) 30-39 degrees; 5) 40-49 degrees; 6) 50 degrees or >

10. dp4 anterior cingulid

0) indistinct; 1) 0/2; 2) low and weakly developed; 3) 2/4; 4) linguolabially well-developed

11. dp4 anteroconid

0) absent; 1) weak; 2) strong

12. dp4 metaconid/protoconid position

0) opposed; 1) protoconid posterior

13. dp4 mesolophid

0) absent; 1) 0/2; 2) weakly developed; 3) 2/4; 4) reaches the lingual side

14. dp4 posterior arm of metaconid (metastylar fold)

0) absent; 1) 0/2; 2) weak and low; 3) 2/4; 4) well-developed and high

15. dp4 metalophulid I

0) absent; 1) 0/2; 2) incomplete, medially interrupted ; 3) 2/4; 4) complete

16. dp4 metalophulid II

0) absent; 1) incomplete, medially interrupted; 2) complete

17. dp4 talonid width

0) as wide as trigonid; 1) slightly wider than trigonid; 2) much wider than trigonid

18. dp4 mesostylid

0) absent; 1) weak; 2) strong

19. dp4 mesoconid

0) absent; 1) 0/2; 2) weak; 3) 2/4; 4) strong

20. dp4 hypoconulid

0) absent; 1) weak; 2) strong

21. dp4 ectolophid

0) absent; 1) anteriorly interrupted (unconnected to protoconid); 2) complete

22. dp4 anterior arm of hypoconid

0) strong; 1) 0/2; 2) weak; 3) 2/4; 4) absent

23. dp4 retention

0) p4 replaces dp4; 1) 0/2; 2) dp4 retained

24. p4 talonid

0) narrow; 1) <= trigonid; 2) slightly wider than trigonid; 3) much wider than trigonid

25. p4 dominant cusps

0) metaconid>protoconid; 1) metaconid=protoconid; 2) protoconid variably>metaconid

26. p4 hypolophid

0) absent; 1) 0/2; 2) present

27. p4 metaconid-protoconid compression

0) absent; 1) present

28. p4 metaconid/protoconid position

0) opposed; 1) protoconid posterior

29. p4 hypoconulid

0) minute to absent; 1) weak; 2) strong

30. p4 mesoconid

0) absent; 1) weak; 2) strong

31. p4 ectolophid

0) absent; 1) 0/2; 2) incomplete, anteriorly interrupted; 3) 2/4; 4) complete, reaches protoconid from mesoconid

32. p4 metalophulid I

0) absent; 1) 0/2; 2) incomplete, unconnected to metaconid; 3) 2/4; 4) complete

33. p4 metalophulid II

0) absent; 1) incomplete, unconnected to metaconid ; 2) complete

34. p4 anterior cingulid

0) absent; 1) 0/2; 2) low, lingually limited

35. p4 posterior arm of metaconid (metastylar fold)

0) absent; 1) weak and low; 2) well-developed and high

36. p4 size

0) p4 >= m1; 1) p4

37. m1 area

0) 0-2 mm2; 1) 0/2; 2) 2-4 mm2; 3) 2/4; 4) 4-6 mm2; 5) 4/6; 6) 6-8 mm2; 7) 6/8; 8) > 8 mm2

38. m1-2 anterolophid (anterocingulid)

0) absent; 1) 0/2; 2) incipient, low

39. m1-2 posterior arm of metaconid

0) absent; 1) 0/2; 2) small (metastylid) ; 3) 2/4; 4) high metastylid (connected to mesostylid)

40. m1-2 anterior arm of molar entoconid

0) absent; 1) 0/2; 2) small; 3) 2/4; 4) high (connected to mesostylid)

41. m1-2 molar paraconid

0) present; 1) reduced; 2) absent

42. m1-2 anteroconid

0) absent; 1) incipient in anterolophid; 2) well-developed

43. m1-2 mesostylid

0) absent; 1) 0/2; 2) weak; 3) 2/4; 4) strong

44. m1-2 ectostylid

0) absent; 1) 0/2; 2) present

45. m1-2 mesoconid

0) small to absent; 1) 0/2; 2) simple cuspid

46. m1-2 ectolophid

0) absent; 1) 0/2; 2) anteriorly incomplete; 3) 2/4; 4) complete, reaches protoconid

47. m1-2 mesolophid

0) absent; 1) variably incipient

48. m1-2 protoconid/metaconid position

0) opposed; 1) 0/2; 2) protoconid posterior

49. m1-2 metaconid/protoconid height

0) protoconid < metaconid; 1) protoconid = metaconid; 2) protoconid > metaconid

50. m1-2 direction of the posterior arm of the protoconid

0) directed toward metaconid; 1) oblique, extending backward

51. m1-2 paralophid-metaconid connection

0) absent; 1) present (metalophulid I)

52. m1-2 metalophulid I

0) complete (ancestral paralophid) ; 1) complex (antero-labial part of paralophid plus lingual part of protolophid); 2) weak to absent; 3) remains of the antero-labial part of paralophid, lingual part of paralophid or neoformation

53. m1-2 metalophulid II (protolophid = posterior arm of protoconid)

0) well-developed ; 1) 0/2; 2) weak; 3) 2/4; 4) small to absent

54. m1-2 hypolophid

0) absent; 1) 0/2; 2) low, interrupted labially; 3) 2/4; 4) well-developed

55. m1-2 anterior arm of hypoconid

0) absent; 1) 0/2; 2) connects weakly to mesoconid; 3) 2/4; 4) strong

56. m1-2 entoconid/hypoconid position

0) opposed; 1) 0/2; 2) entoconid anterior

57. m1-2 hypoconulid

0) strong, inflated; 1) 0/2; 2) 'normal' (simple cuspid) ; 3) 2/4; 4) reduced, submerged into the posterolophid ; 5) 4/6; 6) absent

58. m1-2 entoconid-hypoconid connection by posterolophid

0) present; 1) 0/2; 2) absent

59. m3) hypoconulid

0) forms a transverse third lobe; 1) third lobe narrow; 2) third lobe incorporated into the posterolophid

60. m3) entoconid-hypoconid twinning

0) absent; 1) present

61. molar trigonid height

0) higher than talonid; 1) 0/2; 2) same height as talonid

62. molar trigonid width

0) narrower than talonid; 1) same as talonid

63. size of m3) relative to m2

0) m3 > m2 ; 1) 0/2; 2) m3 = m2 ; 3) 2/4; 4) m3 < m2

64. P2

0) present; 1) absent

65. P3 size/presence

0) present; 1) reduced; 2) absent

66. DP3 size/presence

0) present; 1) reduced; 2) absent

67. dp4 anterocingulum

0) weak; 1) low and mesiodistally widened; 2) high

68. dp4 labial pericingulum

0) present; 1) 0/2; 2) absent

69. dp4 protoloph

0) absent; 1) present

70. dp4 metaconule

0) well developed; 1) 0/2; 2) submerged to the anterior arm of the hypocone; 3) 2/4; 4) indistinct

71. dp4 anterior arm of hypocone

0) absent; 1) present

72. dp4 metaloph

0) connected to metaconule; 1) 0/2; 2) connected on the anterior arm of the hypocone; 3) 2/4; 4) turned posteriorly to join posteroloph; 5) 4/6; 6) submerged into posteroloph (or absent); 7) connected to metaconule and posteroloph; 8) 0/7

73. dp4 endoloph

0) absent; 1) present

74. dp4 mure

0) absent; 1) 0/2; 2) incipient; 3) 2/4; 4) complete

75. dp4 mesolophule

0) absent; 1) 0/2; 2) present

76. dp4 retention

0) P4 replaces DP4; 1) 0/2; 2) DP4 retained

77. P4 size

0) P4>=M1; 1) P4<M1; 2) minute; 3) absent

78. P4 protoconule

0) weak; 1) indistinct

79. P4 metaconule

0) strong; 1) 0/2; 2) weak; 3) 2/4; 4) indistinct

80. P4 protoloph

0) absent; 1) incomplete (lingual protoloph absent); 2) complete

81. P4 metaloph

0) absent; 1) incomplete (lingual metaloph absent); 2) complete;

3) 2/4; 4) submerged in posteroloph; 5) 0/1

82. P4 metacone

0) minute to absent; 1) weak; 2) strong

83. P4 hypocone

0) minute to absent; 1) 0/2; 2) weak; 3) 2/4; 4) strong

84. P4 mesostyle

0) absent; 1) 0/2; 2) weak; 3) 2/4; 4) strong;

85. P4 metacone-paracone compression

0) present; 1) absent

86. P4 anteroloph

0) absent; 1) 0/2; 2) present

87. M1-2 hypocone

0) absent; 1) 0/2; 2) weak; 3) 2/4; 4) strong

88. M1-2 hypocone in relation to protocone

0) more lingual than protocone; 1) 0/2; 2) same level as protocone; 3) 2/4; 4) more labial

89. M1-2 mesostyle

0) absent; 1) 0/2; 2) weak; 3) 2/4; 4) large

90. M1-2 parastyle

0) absent; 1) small; 2) well-developed

91. M1-2 anterostyle

0) absent; 1) 0/2; 2) weak

92. M1-2 enterostyle

0) absent; 1) weak; 2) strong

93. M1-2 anteroloph

0) absent; 1) 0/2; 2) low and isolated from the protocone; 3) 2/4; 4) high and connected to the protocone

94. M1-2 metaconule

0) strong, inflated ; 1) 0/2; 2) reduced; 3) 2/4; 4) minute to indistinct

95. M1-2 metaconule position

0) running onto metacone from protocone; 1) central; 2) submerged into the mure

96. M1-2 metaconule-hypocone connection

0) absent; 1) present

97. M1-2 anterior arm of hypocone

0) absent; 1) weakly developed; 2) well developed, generally strongly connected to the metaconule

98. M1-2 mesolophule

0) absent; 1) 0/2; 2) incipient from metaconule; 3) 2/4; 4) reaches labial side

99. M1-2 protoconule

0) strong, inflated; 1) reduced; 2) submerged in the protoloph to absent

100. M1-2 protoloph connection to protocone

0) strong; 1) 0/2; 2) weak; 3) 2/4; 4) absent

101. M1-2 anteroloph-protoloph connection (with a lingual anteroloph)

0) absent; 1) present

102. M1 metaloph connection

0) connected to metaconule; 1) 0/2; 2) connected on the anterior arm of the hypocone; 3) 2/4; 4) turned posteriorly to join posteroloph; 5) 4/6; 6) submerged into posteroloph (or absent); 7) connected to metaconule and posteroloph; 8) 0/7

103. M2 metaloph connection

0) connected to metaconule; 1) 0/2; 2) connected on the anterior arm of the hypocone; 3) 2/4; 4) turned posteriorly to join posteroloph; 5) 4/6; 6) submerged into posteroloph (or absent); 7) connected to metaconule and posteroloph; 8) 0/7

104. M3 metaloph connection

0) connected to metaconule; 1) 0/2; 2) connected on the anterior arm of the hypocone; 3) 2/4; 4) turned posteriorly to join posteroloph; 5) 4/6; 6) submerged into posteroloph (or absent); 7) connected to metaconule and posteroloph; 8) 0/7

105. M3 angle between protoloph and metaloph

0) 0-30; 1) 0/2; 2) 30-60; 3) 2/4; 4) 60-90; 5) metaloph submerged into posteroloph; 6) turned posteriorly to join posteroloph

106. M1 molar endoloph

0) present; 1) 0/2; 2) absent

107. M2 molar endoloph

0) present; 1) 0/2; 2) absent

108. M3 molar endoloph

0) present; 1) 0/2; 2) absent

109. M1-2 metaconule-protocone connection (lingual metaloph)

0) absent; 1) 0/2; 2) present

110. M1-2 mure

0) absent; 1) 0/2; 2) incipient; 3) 2/4; 4) complete

111. M1-2 pericingulum

0) present; 1) absent

112. M1-2 length/width proportions

0) length > width ; 1) 0/2; 2) length = or < width

113. M1-2 posterior arm of paracone

0) absent; 1) 0/2; 2) weakly developed; 3) 2/4; 4) high (connected to the mesostyle)

114. M1-2 anterior arm of the metacone

0) absent; 1) 0/2; 2) weakly pronounced; 3) 2/4; 4) high (connected to the mesostyle)

115. taeniodont pattern on upper and lower molars

0) absent; 1) present

116. molar cuspids/crests

0) bunodont; 1) 0/2; 2) crests present but low; 3) 2/4; 4) lophodont

117. molar cuspids

0) sharpened; 1) inflated

118. enamel wrinkling on molars

0) absent; 1) present
