# Supplementary material for: Craniodental Morphology and Systematics of a New Family of Hystricognathous Rodents (Gaudeamuridae) from the Late Eocene and Early Oligocene of Egypt
Source: PLoS One. 2011 Feb 22;6(2):e16525. doi: 10.1371/journal.pone.0016525 (PMC3043065; doi:10.1371/journal.pone.0016525)
Supplement: Appendix S2 — Character-taxon matrix employed in phylogenetic analyses. (DOCX) [file pone.0016525.s002.docx]

**Appendix S2**

Modified character-taxon matrix of Marivaux et al. (2004) and Sallam et al. (2009) employed in phylogenetic analyses. Missing and inapplicable data scored as “?”. Polymorphisms were scored as individual character states rather than as, e.g. “0/1” or “1/2”.

[ 1 2 3 4 5]
[ 1234567890 1234567890 1234567890 1234567890 1234567890]

Anadianomys declivis ??2??????? ?????????? ??02120010 4420112020 2010000010
Baluchimys ganeshapher ??2??4???1 1003021002 20???????? ??????2242 2020040010
Baluchimys krabiense ??2114113? ?????????? ?????????? ???????242 2020040010
Birbalomys spp. 1021100002 1104022121 2002120011 4410210142 2020230010
Branisamys luribayensis 112??????? ??4???1120 2002120000 4400108040 2020040010
Bugtimys zafarullahi ?1212511?? ?????????? ??02110000 4022216240 2000040210
Chapattimys wilsoni 1021000014 1022021021 2002120010 4021212042 2020121000
Confiniummys sidiki ?????????0 2100201002 2000100000 0000012222 2110040010
Diamantomys luederitzi 1121221122 2104411021 202??????? ??????8220 2000240211
Eobranisamys romeropittmanae ?????????0 1040421120 10???????? ??????2040 2020040010
Eosallamys paulacoutoi ?????????0 0040421200 2002120000 4020014040 2020040010
**Gaudeamus aegyptius 1121?4???0 0040421231 2302100000 0300?12041 2030040011
Gaudeamus aslius 112??????0 0044421231 2302100000 1310213043 2031040011
Gaudeamus cf. aslius ?12??????? ?????????? ??02110000 4321002043 2030040011
Gaudeamus hylaeus 112??????0 0000421231 2402110000 0310213041 2031040011**Hodsahibia spp. ??21241034 2024221242 2002110001 4021112220 2000141210
Hystrix depereti ?121220140 0044301200 2303120002 4400008040 203004001?
Hystrix primigenia ?12122014? ?????????? ??03120002 4400008040 203004001?
Incamys spp. 1121141150 ?044?21120 2402120000 2210018044 2030040010
Lindsaya derabugtiensis ??2??????4 1103021102 20???????? ??????0220 2020040010
Lophibaluchia spp. ??2??????4 0103011022 20???????? ??????2241 2020040210
Metaphiomys beadnelli 1121161162 1012021001 202??????? ??????4220 2000040211
Ottomania proavita ?????????1 2100021002 2000000000 0001012230 2120041011
Paraphiomys spp. 1121261144 2104001001 202??????? ??????2220 2200040210
”Paraphiomys” simonsi 11???????2 01030?1001 202??????? ??????8220 2201040211
Paraulacodus indicus ??2??????4 1104001101 222??????? ?????18200 2200040210
Petrokozlovia notos 1021?????? ?????????? ?002100122 4010208022 2000020010
Phiocricetomys minutus ?12??????2 1102001002 232??????? ??????0020 2000040121
Phiomys, new species (Quarry I) ?12??????2 1002021002 202??????? ??????0220 2000040201
Phiomys andrewsi 112??????2 1002021002 2002100010 3322010220 2000040201
Phiomys hammudai ?????????1 1023221102 2002110000 4310112020 202004?100
”Phiomys” lavocati 112??????2 1102001002 2002120010 4000?10030 2000040111
”Phiomys aff. paraphiomyoides” ?12??????2 1002021002 202??????? ??????2220 2100040201
Phiomys paraphiomyoides ?12??????2 1002021002 102??????? ??????0220 2000040201
Platypittamys brachyodon 112106116? ?????????? ??01100000 4410010040 2000040010
Protophiomys aegyptensis ??2??????1 1123221122 20???????? ??????2132 2021040010
Protophiomys algeriensis ??21241152 1114321122 2002100100 4311112032 2020040011
Protophiomys durattalahensis ?????????0 1043221102 20???????? ??????2032 202004?011
Protophiomys aff. durattalahensis ?????????? ?????????? ??02110000 4210112032 202004?010
Sacaresia moyaeponsi ???10???52 0100420201 2002220010 3021014200 2040030210
Sallamys pascuali 1121?411?0 0043011200 1002120000 2210014010 2000040010
Talahphiomys lavocati ?????????2 1101001112 20???????? ??????0010 ?01004021?
Talahphiomys libycus ?????????? ?????????? ?????????? ??????0110 ?01004011?
Thryonomys swinderianus 1121440140 1041401200 212??????? ??????8210 2000040011
Tsaganomys altaicus 2121441122 20024220?1 2003120010 4420?18042 2000040010
Waslamys attiai ?12??????1 1023211112 2002110010 4410112032 2020041011
Yuomys cavioides 102??????? ?????????? ??02120110 4410008000 2000040010
New genus and species (Quarry A & E) 112??????2 ?031221102 2001?????? ??????2220 2110040101
New genus and species 1 (L-41) 112??????3 0031121102 2002110010 4222012220 2110040201
New genus and species 2 (L-41) 112??????3 1041221102 2002110000 4221012220 2110041101
New genus and species 3 (L-41) 112??????2 1102021102 2002120010 4200010031 2010040211

[ 1]
[ 5 6 7 8 9 0]
[ 1234567890 1234567890 1234567890 1234567890 1234567890]

Anadianomys declivis 1024402020 21?1?????? ?????01102 12201?4221 2022001020
Baluchimys ganeshapher 1024422?20 2131??1210 061000??31 222012?201 2040112020
Baluchimys krabiense 1014422020 21?1?????? ?????01132 2230122421 2042012420
Birbalomys spp. 1024401220 21011?1100 0610001102 22421?4021 2020000010
Branisamys luribayensis 1004106220 2141?1???? ?????01142 2242124422 2044212424
Bugtimys zafarullahi 1004422020 2101211211 1000201132 2220124441 2042112420
Chapattimys wilsoni 1024400220 21?1?????? ?????01002 1232124021 2020000010
Confiniummys sidiki 1024422020 2121?????? ?????02140 0000104421 2034112420
Diamantomys luederitzi 1044424220 2121112212 140422???? ??????4442 20442?2420
Eobranisamys romeropittmanae 1004206220 2121?????? ?????????? ??????4332 2044212424
Eosallamys paulacoutoi 1004426020 21???????? ?????01142 3220124332 2044212420
**Gaudeamus aegyptius 1044016120 21412????? ?????01141 0231124222 2044212024
Gaudeamus aslius 1024306120 21412????? ?????01141 5230124222 2044212023
Gaudeamus cf. aslius 1024316120 2141?????? ?????????? ?????????? ??????????
Gaudeamus hylaeus 1034006120 21412????? ?????01141 5230124232 2044212024**Hodsahibia spp. 1014421020 2121??1211 1000201102 1222124411 2041112420
Hystrix depereti 1044106120 2140?????? ?????00??2 324412433? ?044212420
Hystrix primigenia 1044406220 2140?????? ?????00??2 324412443? ?044212420
Incamys spp. 1044026220 2141?1???? ?????01141 4144124422 2044212424
Lindsaya derabugtiensis 1024422220 2131??1210 0610001142 2220124220 2040011120
Lophibaluchia spp. 1014422220 2131??1211 14001????? ??????4421 2043012320
Metaphiomys beadnelli 1014422120 2121?12212 140422???? ??????4221 2044212320
Ottomania proavita 1004422220 1121?10210 0310202140 0000104421 ?031112420
Paraphiomys spp. 1044424020 2121222212 140422???? ??????4221 2044212220
”Paraphiomys” simonsi 1044424220 2101?12212 140402???? ??????4211 2040112{24}20
Paraulacodus indicus 104422622? 21?1??2212 160402???? ??????4221 2044212020
Petrokozlovia notos 1044400220 01011?12?? ?????01002 12200?2222 2020000020
Phiocricetomys minutus 10444211-0 21-1?????? ?????????? ?????????? 2?????????
Phiomys, new species (Quarry I) 1034422220 2121?????? ?????????? ??????43?? ?04??1??20
Phiomys andrewsi 1034422220 212120???? ?????01122 2220104321 2044212420
Phiomys hammudai 1014422120 2121?????? ?????01142 2220124421 2043212420
”Phiomys" lavocati 1044421220 2131211211 100002???? ??????420? 2031111020
Phiomys paraphiomyoides 1034422220 2121?????? ?????????? ??????432? 2044212420
”Phiomys aff. paraphiomyoides” 1034422220 2131?????? ?????????? ??????432? 2044212420
Platypittamys brachyodon 1004426220 21412????? ?????01142 4220124421 2044212420
Protophiomys aegyptensis 1024422120 2121??1211 1010001142 2220114421 2044112220
Protophiomys algeriensis 1024422220 21?1??1212 1011201122 12201?4421 2044112220
Protophiomys durattalahensis 1014422020 2121??1211 1703101122 2220124421 2044112320
Protophiomys aff. durattalahensis 1014422020 2121??1211 1400201142 2220124421 2043112420
Sacaresia moyaeponsi 1004404220 2141210212 1610201??2 4044104240 200???2420
Sallamys pascuali 1044426220 2141?1???? ?????01141 4220102402 2044212220
Talahphiomys lavocati 1044421120 214???1211 16000????? ??????4221 2041112020
Talahphiomys libycus 104442112? 21????1211 16001????? ??????4221 2041?12320
Thryonomys swinderianus 1044426220 2131222212 160402???? ??????4300 2034?02020
Tsaganomys altaicus 100440622? 2021212212 00100011?2 2220111402 0044?00020
Waslamys attiai 1014422020 2121?11211 1713201022 2220124421 2041112320
Yuomys cavioides 1044402020 01211????? ?????01102 12001?4231 0200000020
New genus and species (Quarry A & E) 1014422120 213121???? ?????????? ??????4311 2044212420
New genus and species 1 (L-41) 1014422120 2131211211 1704101142 2220104311 2044212420
New genus and species 2 (L-41) 1004422120 2101211111 1704201122 2220124311 2044212420
New genus and species 3 (L-41) 1034422120 2121211011 100302???? ??????4221 2141112020

[ 1 ]
[ 0 1 ]
[ 1234567890 12345678]

Anadianomys declivis 000??00?20 12000200
Baluchimys ganeshapher 0?7??00?00 12330200
Baluchimys krabiense 0000000000 12220200
Birbalomys spp. 0000300020 12200200
Branisamys luribayensis 0474622204 12001400
Bugtimys zafarullahi 0222022202 12400401
Chapattimys wilsoni 0000300020 12200201
Confiniummys sidiki 00?0000000 12100300
Diamantomys luederitzi 04???2??04 12400400
Eobranisamys romeropittmanae 0444622204 12001400
Eosallamys paulacoutoi 0444622204 12000400
**Gaudeamus aegyptius 0666522204 12041400
Gaudeamus aslius 0666522104 12340400
Gaudeamus cf. aslius ?????????? ????0400
Gaudeamus hylaeus 0666522204 12041400**Hodsahibia spp. 0222022201 12320401
Hystrix depereti 1447000204 12000401
Hystrix primigenia 0777000003 12000401
Incamys spp. 0555522204 12201400
Lindsaya derabugtiensis 0000?00000 12220200
Lophibaluchia spp. 0444622200 12220400
Metaphiomys beadnelli 0440022204 12200400
Ottomania proavita 0081002200 12100400
Paraphiomys spp. 0456522204 12200400
”Paraphiomys” simonsi 044??22?04 1220030?
Paraulacodus indicus 0666522204 12400400
Petrokozlovia notos 000??00?00 12000200
Phiocricetomys minutus ?????????? ????0200
Phiomys, new species (Quarry I) 0???????0? 12??0300
Phiomys andrewsi 077??22?04 12200300
Phiomys hammudai 0770422204 12100101
”Phiomys” lavocati 06???2??00 12200200
Phiomys paraphiomyoides 077??22?04 12200300
”Phiomys aff. paraphiomyoides” 077??22?04 12??0300
Platypittamys brachyodon 0222022204 12000400
Protophiomys aegyptensis 0000421102 12220301
Protophiomys algeriensis 0000412200 12220300
Protophiomys durattalahensis 0000421003 12230300
Protophiomys aff. durattalahensis 0000422003 12230300
Sacaresia moyaeponsi 0666500000 02110400
Sallamys pascuali 0472022204 10000400
Talahphiomys lavocati 0440422000 12100200
Talahphiomys libycus 0650421000 12220200
Thryonomys swinderianus 06446222?4 12000400
Tsaganomys altaicus 0000000000 12000400
Waslamys attiai 0700320001 12230301
Yuomys cavioides 0000000000 12000400
New genus and species (Quarry A & E) 0770421104 12100100
New genus and species 1 (L-41) 0777422204 12100300
New genus and species 2 (L-41) 0770422004 02100101
New genus and species 3 (L-41) 006??22?04 12220200
